# Study on association factors of intestinal infectious diseases based-Bayesian spatio-temporal model

**DOI:** 10.1186/s12879-023-08665-3

**Published:** 2023-10-24

**Authors:** Yancen Zhan, Hua Gu, Xiuyang Li

**Affiliations:** 1 Department of Big Data in Health Sciences, and Center for Clinical Big Data and Statistics, Second Affiliated Hospital, College of Medicine, Zhejiang University, Hangzhou, 310058 Zhejiang China; 2 The Cancer Hospital, University of Chinese Academy of Sciences (Zhejiang Cancer Hospital), Institute of Basic Medicine and Cancer, Chinese Academy of Sciences, Hangzhou, 310022 China

**Keywords:** Intestinal infectious diseases, Spatio-temporal model, Bayesian framework

## Abstract

**Background:**

Intestinal infectious diseases (IIDs) are a significant public health issue in China, and the incidence and distribution of IIDs vary greatly by region and are affected by various factors. This study aims to describe the spatio-temporal trends of IIDs in the Chinese mainland and investigate the association between socioeconomic and meteorological factors with IIDs.

**Methods:**

In this study, IIDs in mainland China from 2006 to 2017 was analyzed using data obtained from the China Center for Disease Control and Prevention. Spatio-temporal mapping techniques was employed to visualize the spatial and temporal distribution of IIDs. Additionally, mean center and standard deviational ellipse analyses were utilized to examine the spatial trends of IIDs. To investigate the potential associations between IIDs and meteorological and socioeconomic variables, spatiotemporal zero-inflated Poisson and negative binomial models was employed within a Bayesian framework.

**Results:**

During the study period, the occurrence of most IIDs has dramatically reduced, with uneven reductions in different diseases. Significant regional differences were found among IIDs and influential factors. Overall, the access rate to harmless sanitary toilets (ARHST) was positively associated with the risk of cholera (RR: 1.73, 95%CI: 1.08-2.83), bacillary dysentery (RR: 1.32, 95%CI: 1.06-1.63), and other intestinal infectious diseases (RR: 1.88, 95%CI: 1.52-2.36), and negatively associated with typhoid fever (RR: 0.66, 95%CI: 0.51-0.92), paratyphoid fever (RR: 0.71, 95%CI: 0.55-0.92). Urbanization is only associated with hepatitis E (RR: 2.48, 95%CI: 1.12-5.72). And GDP was negatively correlated with paratyphoid fever (RR: 0.82, 95%CI: 0.70-0.97), and bacillary dysentery (RR: 0.77, 95%CI: 0.68-0.88), and hepatitis A (RR: 0.84, 95%CI: 0.73-0.97). Humidity showed positive correlation with some IIDs except for amoebic dysentery (RR: 1.64, 95%CI: 1.23-2.17), while wind speed showed a negative correlation with most IIDs. High precipitation was associated with an increased risk of typhoid fever (RR: 1.52, 95%CI: 1.09-2.13), and high temperature was associated with an increased risk of typhoid fever (RR: 2.82, 95%CI: 2.06-3.89), paratyphoid fever (RR: 2.79, 95%CI: 2.02-3.90), and HMFD (RR: 1.34, 95%CI: 1.01-1.77).

**Conclusions:**

This research systematically and quantitatively studied the effect of socioeconomic and meteorological factors on IIDs, which provided causal clues for future studies and guided government planning.

## Background

Intestinal infectious diseases (IIDs) are common in China. According to the Chinese Center for Disease Control and Prevention, the incidence of intestinal infectious diseases in China was around 100 per 100,000 population in 2006 and around 240 per 100,000 population in 2017 [1], causing a considerable disease burden.

During our study period, the change of occurrences in IIDs accompanied by the promotion of harmless sanitary, the upsurge of economics, and the change of climate under the background of global warming. Domestically, there are distinct regional differences in climate, environmental conditions and urbanization and variation in infectious diseases is to be expected across the country. Previous studies have assessed the connection between these factors and the morbidity of IIDs. For example, Chen et al. found that sanitary toilet use had an impact on IIDs in Jiangsu Province in China [2]. Li et al. identified the association between meteorological factors and bacillary dysentery in Beijing [3]. However, those studies were limited to a single disease and local areas and lacked geographical integrity and disease comprehensiveness. Thus, we intended to conduct systematic and national research to find out how these factors affected the occurrence of IIDs.

Addressing this issue requires a comprehensive approach that takes into account regional differences and factors that contribute to the occurrence of IIDs. To achieve our goals, we applied the Bayesian hierarchical spatio-temporal model. Our datasets contained various scales of spatial and temporal variability and involved many observation locations, which could limit the effectiveness of traditional general regression models [4]. Therefore, the model with space, time, and space-time interaction structure was needed. The Bayesian hierarchical spatio-temporal models not only fulfilled the requirements but provided simple strategies for incorporating complicated hierarchy within the Markov chain Monte Carlo framework. Several studies have applied this model to disease surveillance datasets. For instance, Cao et al. used the spatio-temporal model to analyze the influential factor for tuberculosis in mainland China [5]. Liu et al. applied Bayesian spatio-temporal analysis to study the association of air pollutants with tuberculosis in Hubei Province in China [6].

In this study, we aim to describe the spatio-temporal trends of IIDs in the Chinese mainland and investigate the association between sanitary, socioeconomic, and meteorological factors with IIDs. Thus, to better understand the pattern of IIDs and guide the strategic direction of government prevention.

## Methods

### Definition of intestinal infectious diseases

Intestinal infectious diseases considered in this study included bacterial, viral, and protozoan infections, according to the International Classification of Diseases 11^th^ Revision and the Law of the People's Republic of China on the Prevention and Treatment of Infectious Diseases. They are cholera, bacillary dysentery, typhoid, paratyphoid, amoebic dysentery, hepatitis A (HAV), hepatitis E (HEV), hand, foot, and mouth disease (HFMD), and other infectious intestinal diseases (OIIDs). These diseases are monitored by the national surveillance system and classified into 3 classes: A, B, and C in terms of severity. Cholera is a class A infectious disease, and bacillary dysentery, typhoid, paratyphoid, amoebic dysentery, hepatitis A, and hepatitis E are in class B.

### Data collection

Annually reported intestinal infectious diseases in 31 provinces of mainland China from 2006 to 2017 were collected from the public health science data center issued by the Chinese Center for Disease Control and Prevention (https://www.phsciencedata.cn/Share/en/). However, due to the limitation of this dataset, we only study 30 provinces in China and Tibet, Taiwan, Hongkong, and Macao are excluded.

The socio-economic variables considered in this study are access rate to harmless sanitary toilets (ARHST), gross domestic product (GDP), and urbanization. Harmless sanitary toilets are referred to as those that met the basic requirements of sanitary toilets and have facilities for the decontamination of feces [2]. These variables and the total population from 2006 to 2017 were collected from the Chinese National Bureau of Statistics (http://www.stats.gov.cn/), and ARHST was provided by National Health Commission.

The meteorological variables, including temperature, precipitation, wind speed, and humidity, were obtained from Global Historical Climatology Network-Daily (Version 3) in US National Centers for Environmental Information (https://www.ncei.noaa.gov). We applied Inverse Distance Weighting to preprocess the data, then we computed annual averages for each province.

Chinese map was obtained from the National Catalogue Service For Geographic Information (https://www.webmap.cn/main.do?method=index).

### Mean center and standard deviational ellipse

The mean center serves as a valuable metric in spatial analysis as it represents the average x and y coordinates of all the features within the study area. The standard deviational ellipse provides insights into the elongation and orientation of the feature distribution, by calculating the standard deviation of both the x and y coordinates from the mean center, subsequently defining the axis of the ellipse. Both methods visually assess the degree of elongation and directional characteristics exhibited by the feature distribution, thereby aiding in the interpretation of spatial patterns and trends.

The mean center is computed as:

$$\overline X=\frac{\sum_{i=1}^nx_i}n,\;\overline Y=\frac{\sum_{i=1}^ny_i}n$$

The standard deviational ellipse is calculated as:

$$\begin{array}{c}SDE_x=\sqrt{\frac{\sum_{i=1}^n\;\left(x_1-\overline X\right)^2}n}\\SDE_y=\sqrt{\frac{\sum_{i=1}^n\;\left(y_1-\overline Y\right)^2}n}\end{array}$$

Where ${x}_{i}$ and ${y}_{i}$ are the coordinates for feature $i$, and $n$ is equal to the total number of features.

### Statistical model

#### Zero-inflated model

The cholera dataset had excess zeros, as stated by Lambert, which might lead to biased parameter estimates and misleading inference if we failed to account for them [7]. To tackle this problem, we chose the zero-inflated Poisson (ZIP) model which can effectively address the presence of excess zeros [8]. ZIP has a logit section and Poisson section, and the structure of this model can be proposed as follows:

$$P\left({y}_{ij}\right)=\left\{\begin{array}{c}{\pi }_{ij}+\left(1-{\pi }_{ij}\right){e}^{-{\mu }_{ij}} {y}_{ij}=0\\ \left(1-{\pi }_{ij}\right)\frac{{e}^{-{\mu }_{ij}}{{\mu }_{ij}}^{{y}_{ij}}}{{y}_{ij}!} {y}_{ij}\ge 1\end{array}\right.$$

where ${y}_{ij}$ denotes the number of observed cases in district $i$($i=\mathrm{1,2},\dots ,30$), year $j(j=\mathrm{1,2},\dots ,12)$; ${\pi }_{ij}$ denotes the probability of a zero occurrence in district $i$, year $j$; ${\mu }_{ij}$ denotes the parameter of Poisson distribution which is the expected number of cases in district $i$, year $j$. Due to the lack of pre-designated truncated Poisson distribution in WinBUGS software, we applied the mixing probability hurdle model [9] and “zeros trick” [10] to fit the ZIP model. Under the mentioned assumptions, the logarithmic transformation of ${\mu }_{ij}$ can be modeled as:

$$logit\left({\pi }_{ij}\right)={\alpha }_{0}+{v}_{i}+{u}_{i}+{g}_{j}+{psi}_{ij}$$

$${\mu }_{ij}={e}_{ij}*{rr}_{ij}$$

$$\mathrm{ln}\left({\mu }_{ij}\right)={\mathrm{ln}\left({e}_{ij}\right)+\alpha }_{0}+{v}_{i}+{u}_{i}+{g}_{j}+{psi}_{ij}$$

where ${e}_{ij}$ denoted the standardized number of cases in district $i$($i=\mathrm{1,2},\dots ,30$), year $j(j=\mathrm{1,2},\dots ,12)$; ${rr}_{ij}$ denoted the relative risk in district *i*, year *j*; ${\alpha }_{0}$ denoted intercept; ${v}_{i}$ denoted the unstructured spatial random effect; ${u}_{i}$ denoted the spatial effect and accounted for spatial correlation; ${g}_{j}$ denoted the time tendency effect; and ${psi}_{ij}$ denoted the spatio-temporal interactive effect.

#### Negative binomial model

For the rest of the diseases in this study, the data exhibit overdispersion in which the variance exceeds the mean, failing to meet the requirement of Poisson distribution. Thus, we chose the negative binomial distribution as the base distribution for our Bayesian spatio-temporal hierarchy model. The structure of this model is defined as follows:

$${y}_{ij} \sim Poisson\left({\lambda }_{ij}\right), {\lambda }_{ij}\sim gamma\left({r}_{ij},{b}_{ij}\right)$$

$${\mu }_{ij}=\frac{{r}_{ij}}{{b}_{ij}}, {\mu }_{ij}={e}_{ij}*{rr}_{ij}$$

$$\mathrm{ln}\left({\mu }_{ij}\right)={\mathrm{ln}\left({e}_{ij}\right)+\alpha }_{0}+{v}_{i}+{u}_{i}+{g}_{j}+{psi}_{ij}$$

where ${\lambda }_{ij}(i=\mathrm{1,2},\dots ,30; j=\mathrm{1,2},\dots ,12)$ is the mean of the Poisson distribution that follows Gamma distribution. The rest of the denotations are the same as mentioned above.

#### Model computation

To estimate the posterior distribution under the Bayesian methodology, we specified different prior distributions for the parameters respectively. Specifically, we applied a conditional autoregressive model for ${u}_{i}$ and defined an adjacency matrix to reflect the spatial relationships. For ${g}_{j}$, we specified first-order autoregressive prior.

After building up the frame of these models, we introduced independent variables which we standardized to avoid numerical problems in the computation process of the model [11]. We used the Markov chain Monte Carlo (MCMC) algorithm to estimate parameters. To monitor the convergence of the MCMC chain during the iteration, we applied a trace history plot, autocorrelation plot, and density plot. If trace history levels out, autocorrelation turns to zero, and the density plot shows the normal distribution, iteration tends to be stable.

Before constructing the model, we conducted VIF calculations for all candidate variables to assess the presence of multicollinearity. Variables with VIF values below 10 were selected for inclusion in the model [12].

The disease mapping, mean center analysis, and standard deviational ellipse analysis were performed using ArcGIS software (version 10.7, ESRI Inc.; Redlands, CA, USA). The statistical model development and analysis in this study were conducted using WinBUGS (version 1.4.3, MRC Biostatistics Unit, Cambridge Biomedical Campus, Cambridge Institute of Public Health, Forvie Site, Robinson Way, Cambridge CB2 0SR, UK). The results were organized and tabulated using Microsoft Excel software. The Relative Risk and confidence intervals were plotted using R software (version 4.2.2). Additionally, the VIF values were computed using R software (version 4.2.2). The differences were considered to be statistically significant at *P*-value ≤ 0.05 with the two-sided test.

## Results

### Descriptive results

Overall, 32,548,000 IIDs cases were reported in China from 2006 to 2017, and the annual national incidences of IIDs changed from 56.3898/100,000 in 2006 to 93.1011/100,000 in 2019. According to the descriptive summary, HFMD was the most common intestinal infectious disease. The total incidence rates and number of cases of IIDs in China from 2006-2017 (1/100,000) are showed in Table 1.

**Table 1:** The total incidence rates and number of cases of IIDs in China from 2006-2017 (1/100,000)

**year**	**Cholera**		**Typhoid fever**		**Paratyphoid fever**		**Bacillary dysentery**		**Amoebic dysentery**		**Hepatitis A**		**Hepatitis E**		**HMFD**		**OIIDs**	
	**Incidence**	**Case**	**Incidence**	**Case**	**Incidence**	**Case**	**Incidence**	**Case**	**Incidence**	**Case**	**Incidence**	**Case**	**Incidence**	**Case**	**Incidence**	**Case**	**Incidence**	**Case**
**2006**	0.0122	158	1.2479	16,308	0.7395	9,668	32.1029	417,199	0.2576	3,362	5.2515	68,357	1.4536	19,005	1.0424	13,630	56.3898	737,030
**2007**	0.0125	164	0.9846	12,941	0.5695	7,486	27.7380	362,744	0.2565	3,369	5.8681	76,780	1.5654	20,577	6.3405	83,333	56.6473	744,504
**2008**	0.0127	168	0.8284	10,946	0.3553	4,671	23.4330	308,466	0.2198	2,903	4.2422	55,541	1.4020	18,525	37.0059	488,935	55.3019	730,647
**2009**	0.0064	85	0.9358	12,402	0.3397	4,504	20.2782	267,394	0.1696	2,250	3.3012	43,438	1.5267	20,268	87.0111	1,155,122	49.3711	655,582
**2010**	0.0118	85	0.7279	9,713	0.3240	4,325	18.7347	249,091	0.1640	2,187	2.6430	35,188	1.7743	23,682	132.9599	1,772,720	55.9323	746,516
**2011**	0.0018	157	0.6539	8,768	0.2260	3,030	17.6392	235,073	0.1200	1,605	2.3459	31,375	2.1778	29,199	120.7916	1,619,434	62.3898	836,525
**2012**	0.0056	24	0.6543	8,816	0.2362	3,182	15.2872	204,696	0.1081	1,457	1.8149	24,281	2.0240	27,270	160.9632	2,167,433	65.7348	1,012,552
**2013**	0.0039	53	0.7919	10,722	0.2521	3,414	13.8329	186,427	0.1009	1,364	1.6428	22,001	2.0606	27,901	135.0312	1,827,834	74.7828	1,012,552
**2014**	0.0018	24	0.7719	10,459	0.2441	3,308	11.2404	151,481	0.0932	1,261	1.9163	25,786	1.9915	26,984	205.0565	2,777,783	64.0175	867,498
**2015**	0.0010	13	0.6491	8,843	0.2050	2,793	10.1089	136,990	0.0871	1,187	1.6637	22,414	1.9941	27,165	146.5996	1,995,356	68.8175	937,542
**2016**	0.0020	27	0.6265	8,587	0.1686	2,311	8.9045	121,396	0.0891	1,221	1.5528	21,208	2.0369	27,919	178.1562	2,440,443	74.3082	1,018,536
**2017**	0.0010	14	0.6359	8,773	0.1462	2,017	7.8504	107,634	0.0757	1,045	1.3679	18,774	2.1027	29,006	139.8389	1,928,576	93.1011	1,284,512

### Spatial and temporal distribution of IIDs

#### Cholera

**Fig. 1:**
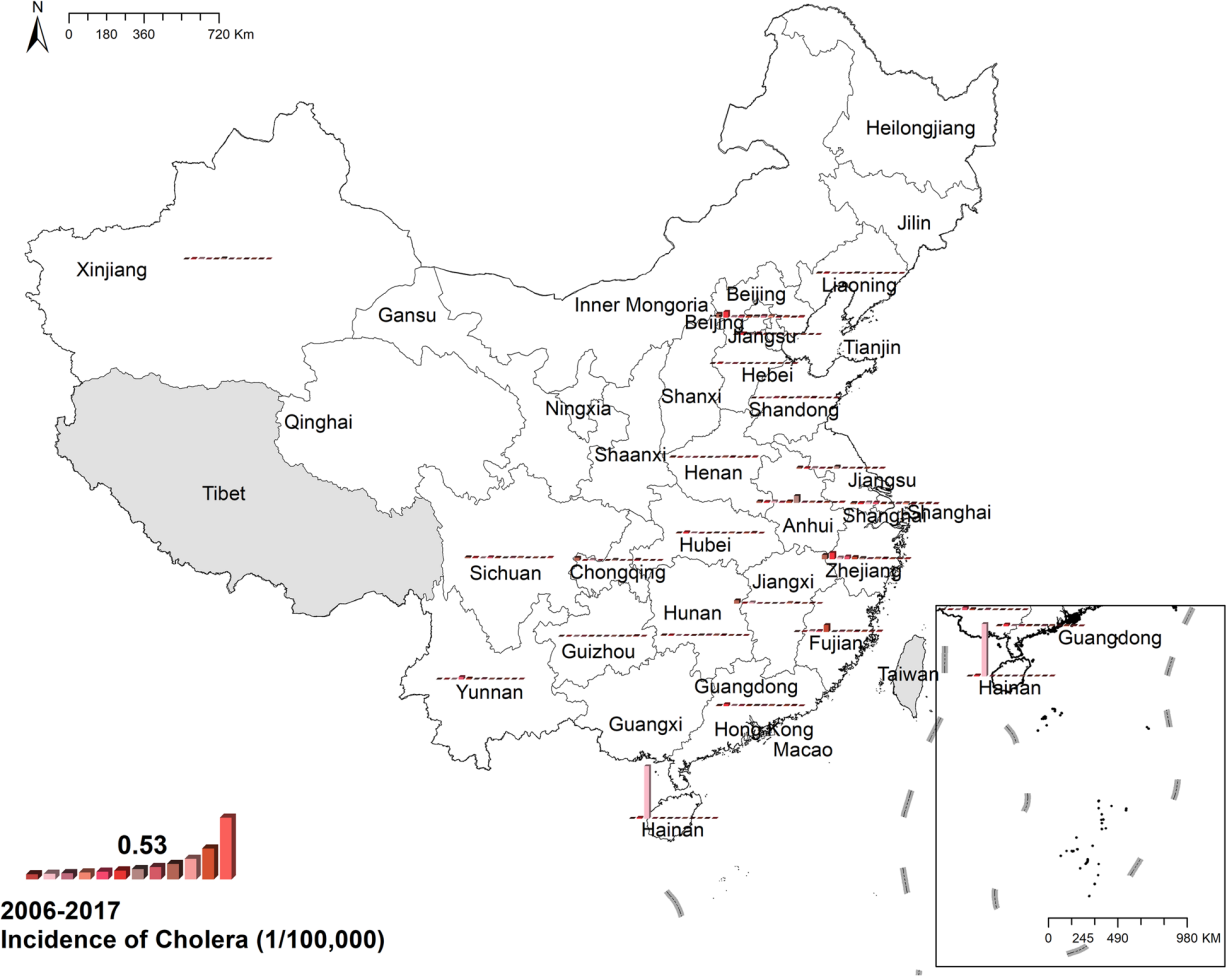
Spatial and temporal distribution of cholera

The national prevalence of cholera fell from 0.012/100,000 in 2006 to 0.001/100,000 in 2017. Most cases were concentrated in coastal provinces, like Zhejiang, Fujian, and Hainan, while in central China, there was a mass of zero cases. During the study period, the highest number of reported cases was 89 in Hainan in 2008. Figure 1 Spatio-temporal distribution of cholera.

#### Typhoid fever

**Fig. 2:**
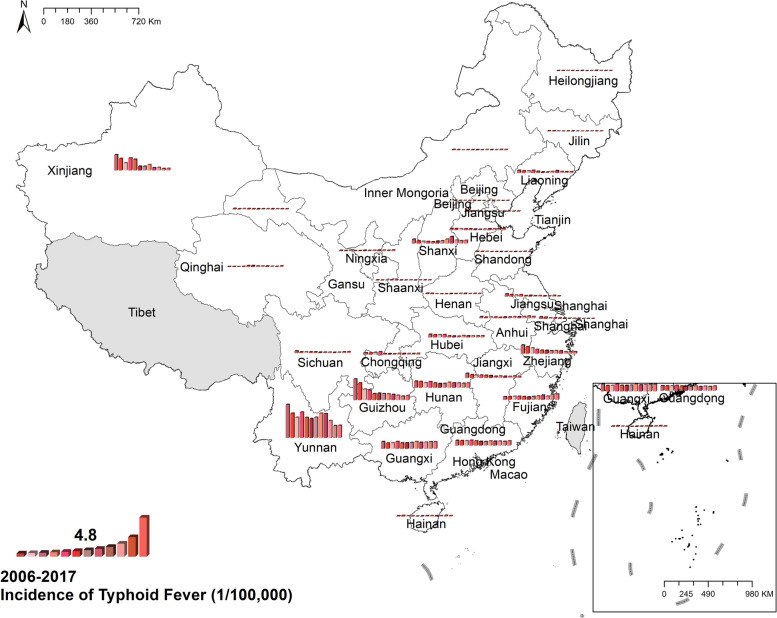
Spatial and temporal distribution of typhoid fever

The national prevalence of typhoid fell from 1.25 cases per 100,000 in 2006 to 0.64 cases per 100,000 in 2017. The spatial distribution of typhoid showed a difference between southern and northern China, in which the morbidity in the south was higher than in the north. Yunnan was the province with the highest typhoid prevalence in China, with 9.66 cases per 100,000 in 2006 and 3.59 cases per 100,000 in 2017. Figure 2 Spatio-temporal distribution of typhoid fever.

#### Paratyphoid fever

**Fig. 3:**
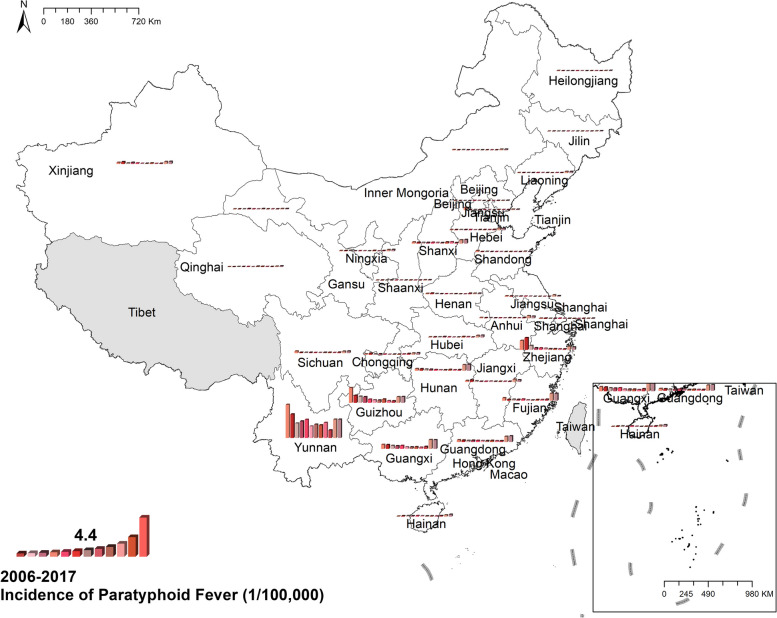
Spatial and temporal distribution of paratyphoid fever

The national prevalence of paratyphoid decreased from 0.74 cases per 100,000 in 2006 to 0.15 cases per 100,000 in 2017, however, the morbidity of several provinces increased in 2016 and 2017, like Yunnan, Guangxi, Guangdong, and Fujian. The occurrence of paratyphoid concentrated in southern China, especially Yunnan, the province with the highest paratyphoid prevalence, with 8.71 cases per 100,000 in 2006 and 4.81 cases per 100,000 in 2017. Figure 3 Spatio-temporal distribution of paratyphoid fever.

#### Bacillary dysentery

**Fig. 4:**
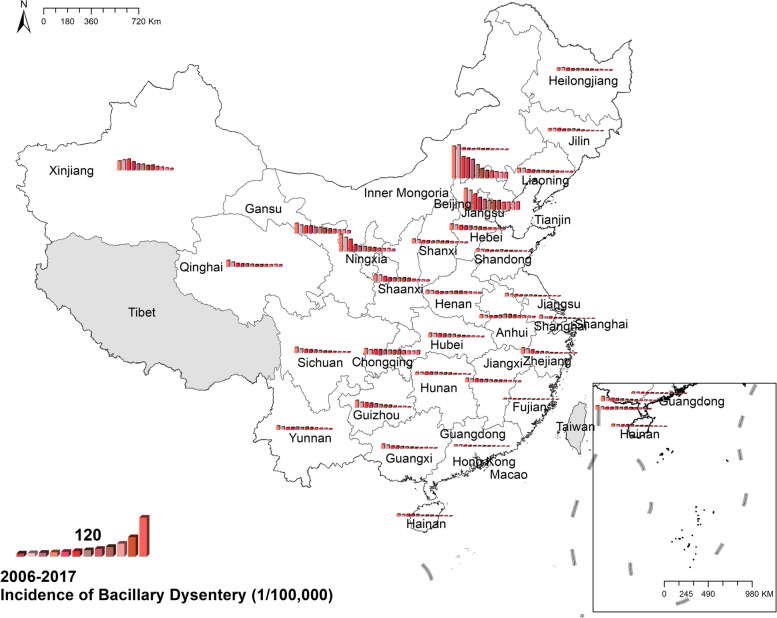
Spatial and temporal distribution of bacillary dysentery

The national prevalence of bacillary dysentery fell from 32.10 cases per 100,000 in 2006 to 7.85 cases per 100,000 in 2017, a clear reduction in occurrence over time as it showed in Fig 4. Spatially, the morbidity in north-western China was higher than in south-eastern provinces. Overall, Beijing and Tianjin were the two provinces with the highest morbidity of bacillary dysentery in China. In 2006, the morbidity in Beijing, 226.82 cases per 100,000, was the highest in China, and in 2017 the highest was Tianjin with 53.66 cases per 100,000 residents. Figure 4 Spatio-temporal distribution of bacillary dysentery.

#### Amoebic dysentery

**Fig. 5:**
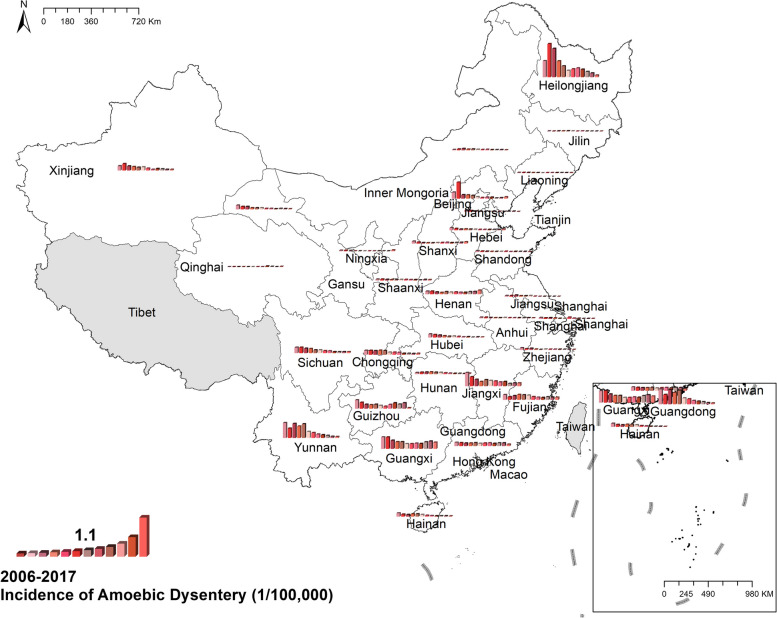
Spatial and temporal distribution of amoebic dysentery

The national prevalence of amoebic dysentery decreased from 0.26 cases per 100,000 in 2006 to 0.08 cases per 100,000 in 2017. Spatially, southern China had more cases than northern China, except for Heilongjiang, in which the morbidity reached 2.16 cases per 100,000 was the highest during the study period. Figure 5 Spatio-temporal distribution of amoebic dysentery.

#### Hepatitis A

**Fig. 6:**
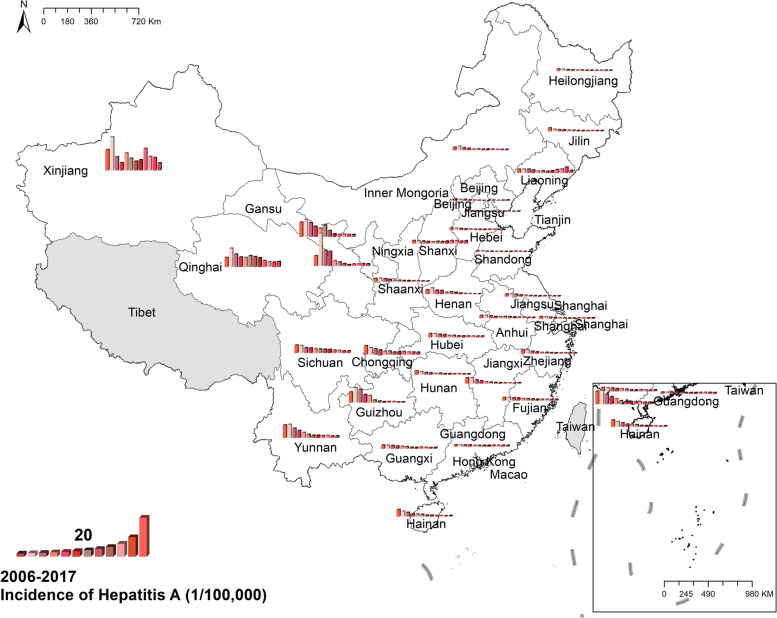
Spatial and temporal distribution of hepatitis A

The national prevalence of hepatitis A fell from 5.25 cases per 100,000 in 2006 to 1.37 cases per 100,000 in 2017. The preponderance of hepatitis A cases was in north-western China, like Xinjiang, Gansu, and Qinghai. In Xinjiang, the morbidity was 16.52 cases per 100,000 in 2006 and 8.75 cases per 100,000 in 2017, both of which were the highest in China. Figure 6 Spatio-temporal distribution of hepatitis A.

#### Hepatitis E

**Fig. 7:**
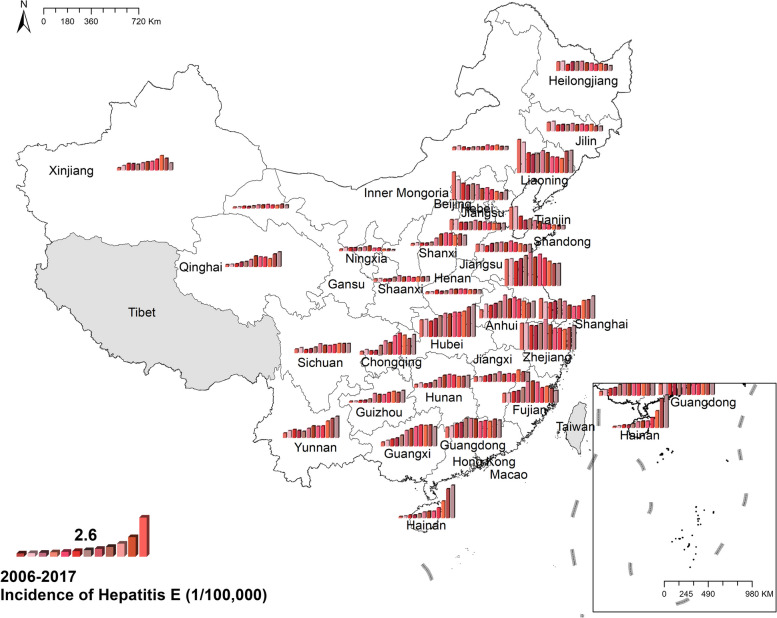
Spatial and temporal distribution of hepatitis E

Unlike other enteric infectious diseases, the national prevalence of hepatitis E increased from 1.45 cases per 100,000 in 2006 to 2.10 cases per 100,000 in 2017. Hainan witnessed the quickest increase of hepatitis E, while a few provinces like Beijing and Tianjin experienced a decline of hepatitis E. Spatially, eastern provinces had a higher incidence than western and central provinces. The highest morbidity was in Jiangsu, with 4.12 cases per 100,000 in 2006 and 3.48 cases per 100,000 in 2017. Figure 7 Spatio-temporal distribution of hepatitis E.

#### Hand, Foot, and Mouth Disease (HFMD)

**Fig. 8:**
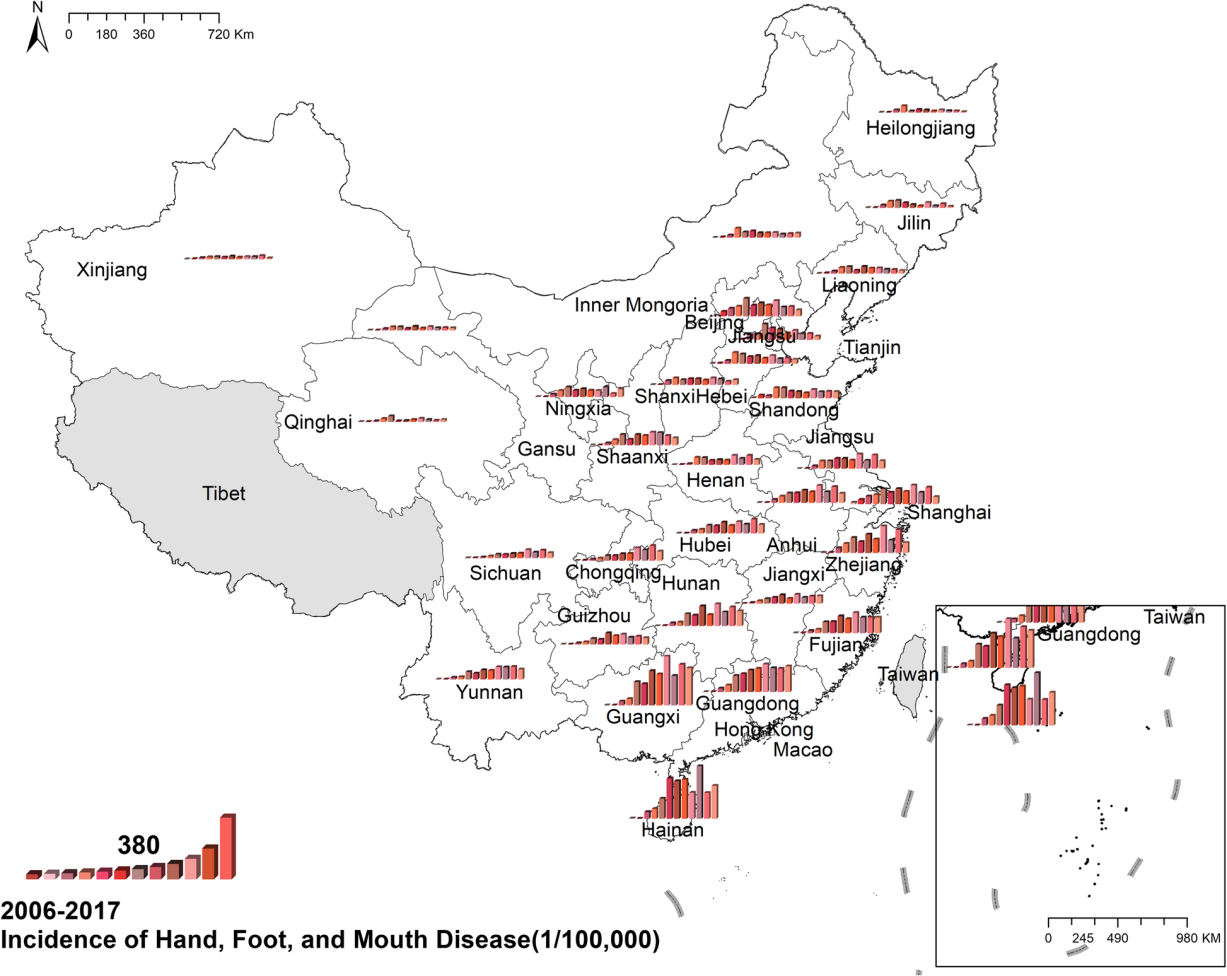
Spatial and temporal distribution of hand, foot, and mouth disease

The national prevalence of hand, foot, and mouth disease increased from 1.04 cases per 100,000 in 2006 to 139.84 cases per 100,000 in 2017. Generally, south-eastern China had more cases than north-western China. Figure 8 Spatio-temporal distribution of hand, foot, and mouth disease.

#### Other infectious intestinal diseases

**Fig. 9:**
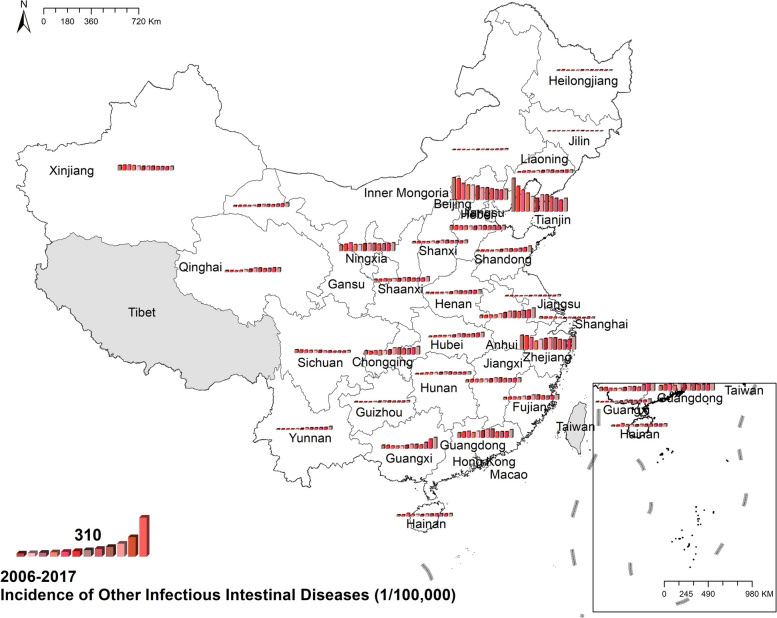
Spatial and temporal distribution of other infectious intestinal diseases

The national prevalence of OIIDs increased from 56.39 cases per 100,000 in 2006 to 93.10 cases per 100,000 in 2017. The preponderance of other infectious intestinal diseases was in Beijing, Tianjin, and Zhejiang. For the rest provinces, the spatial distribution exhibited evenly in Fig. 9. Figure 9 Spatio-temporal distribution of other infectious intestinal diseases.

### Spatial and temporal distribution of covariables

#### Socioeconomic variables

**Fig. 10:**
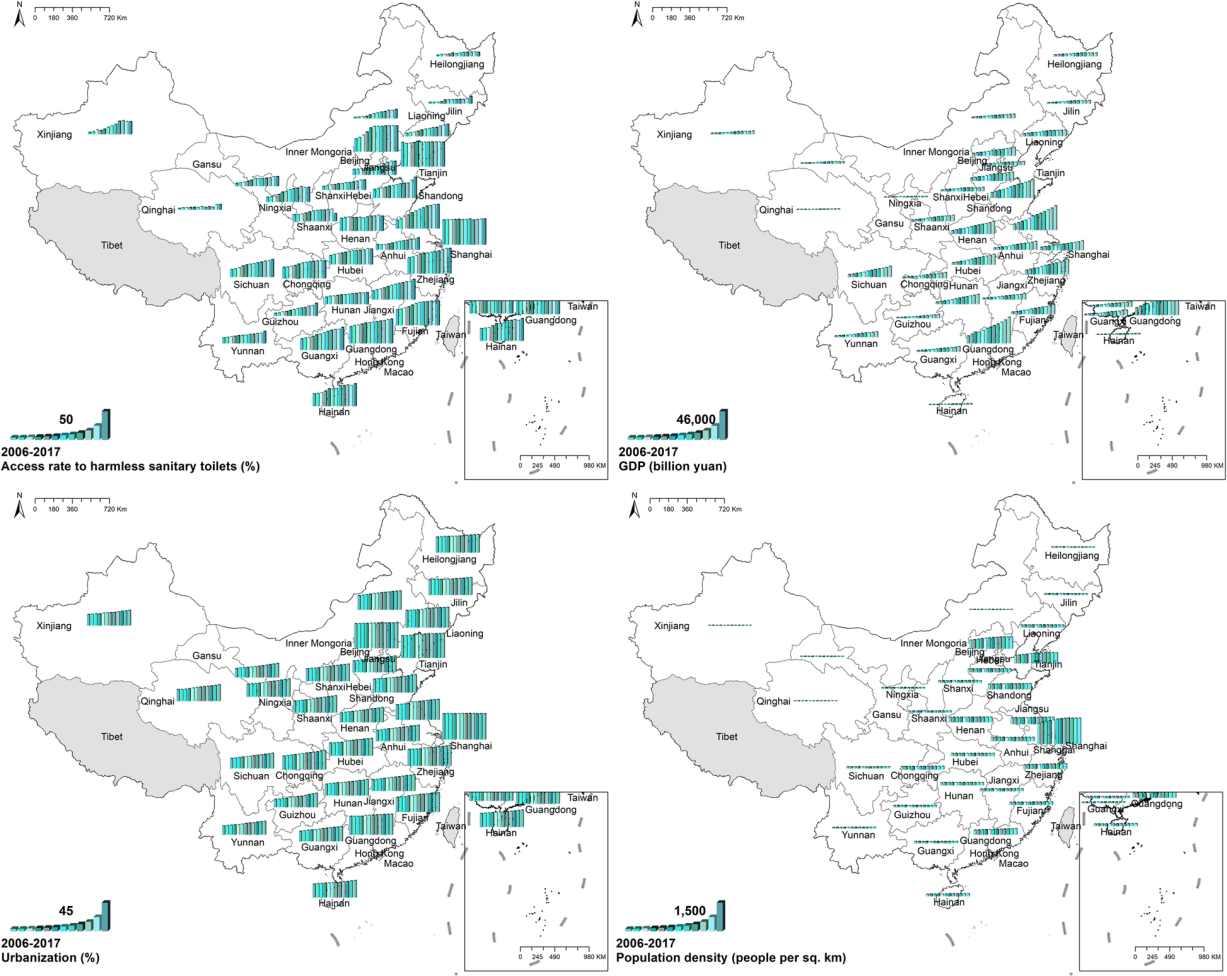
Spatial and temporal distribution of socioeconomic variables

During the study period, national ARHST increased from 32.30% in 2006 to 62.7% in 2017. Spatially, ARHST in south-eastern China was higher than in north-western provinces. Shanghai owned the highest ARHST while Qinghai had the lowest ARHST. The national GDP increased from 219,028.5 billion yuan in 2016 to 830,945.7 billion yuan in 2017. Spatially, south-eastern provinces had higher GDP than north-western and central provinces. There was an upsurge in national urbanization as well, from 44.34% in 2006 to 60.24% in 2017. Spatially, urbanization in eastern provinces was higher than in western provinces. Figure 10 Spatio-temporal distribution of socioeconomic variables.

#### Meteorological variables

**Fig. 11:**
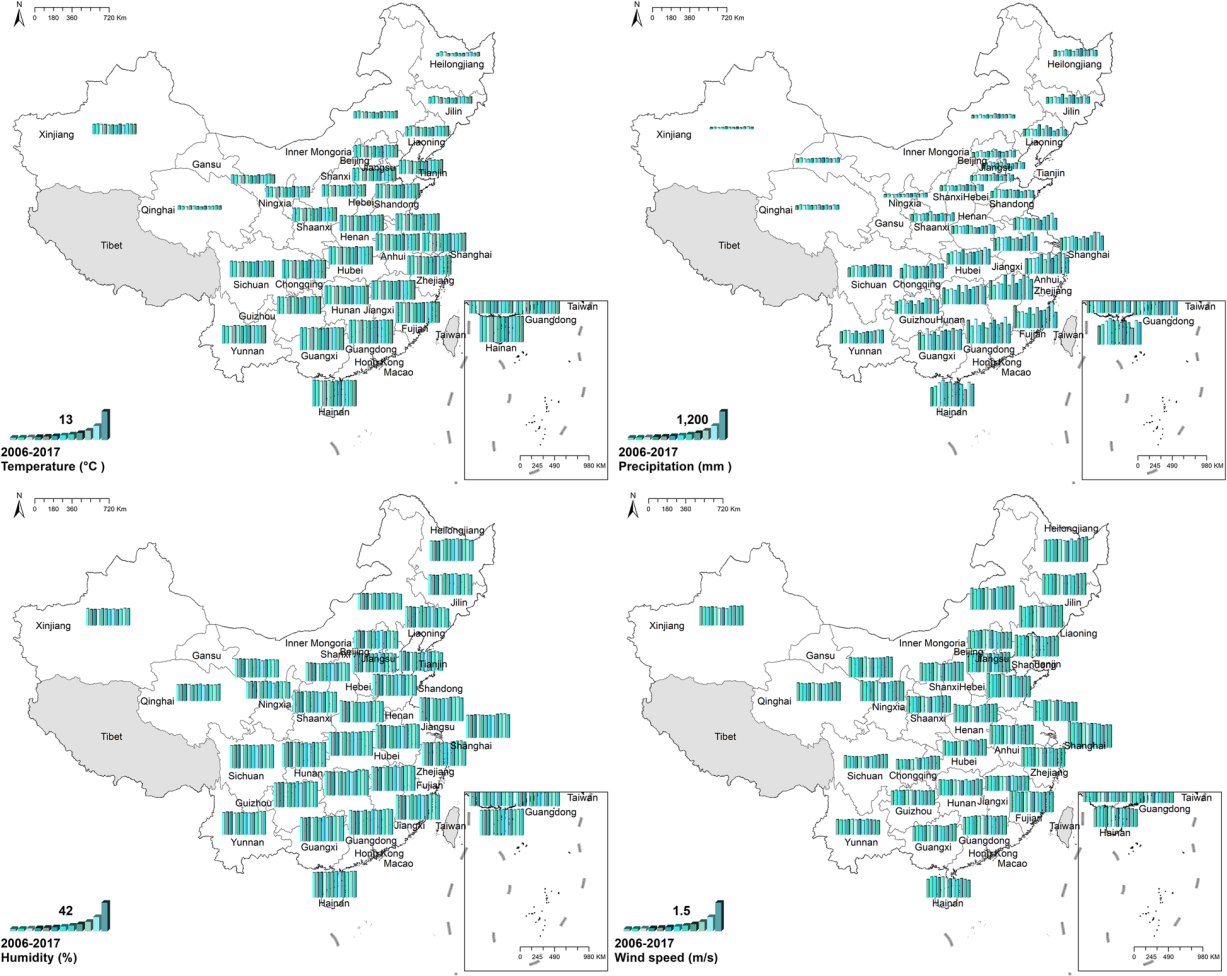
Spatial and temporal distribution of meteorological variables

For meteorological variables, there was no evident temporal change. However, the regional distribution of meteorological variables was uneven. For temperature and precipitation, southern China was higher than northern China. Precipitation in south-eastern and central provinces was higher than in the north-western. There were differences in wind speed between the central provinces and the rest. Figure 11 Spatio-temporal distribution of meteorological variables.

### Spatio-temporal evolution of IIDs

#### Cholera

**Fig. 12:**
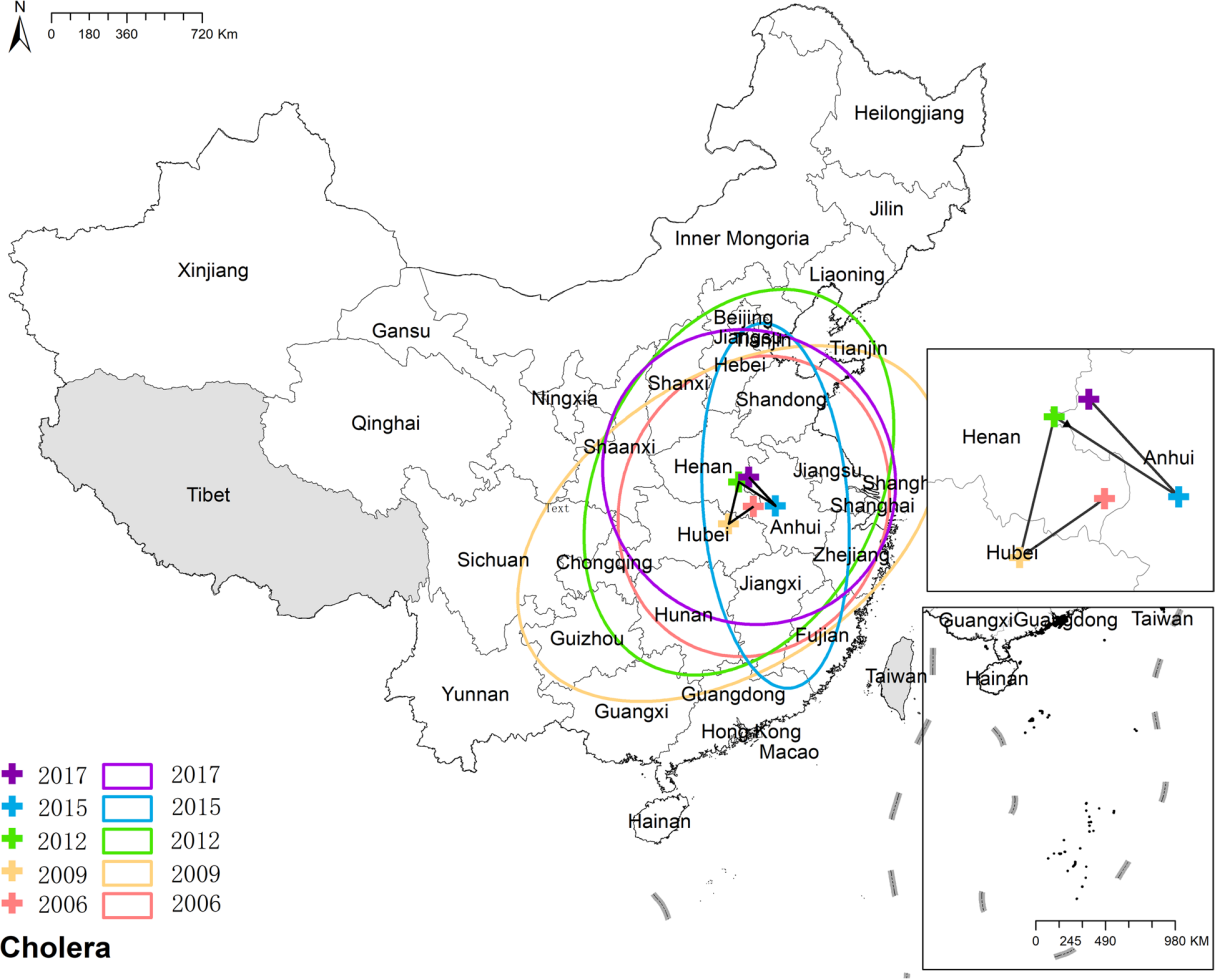
The results of mean center and standard deviational ellipse of cholera

During the research period, the mean center of cholera remained concentrated in central China, specifically in Anhui, Hubei, and Henan provinces. As for the change in the ellipse representing the distribution, it exhibited various alterations without a predominant trend, suggesting that the spatial distribution of cholera was characterized by fluctuations and no consistent directional pattern was observed. Figure 12 the results of mean center and standard deviational ellipse of cholera.

#### Typhoid fever

**Fig. 13:**
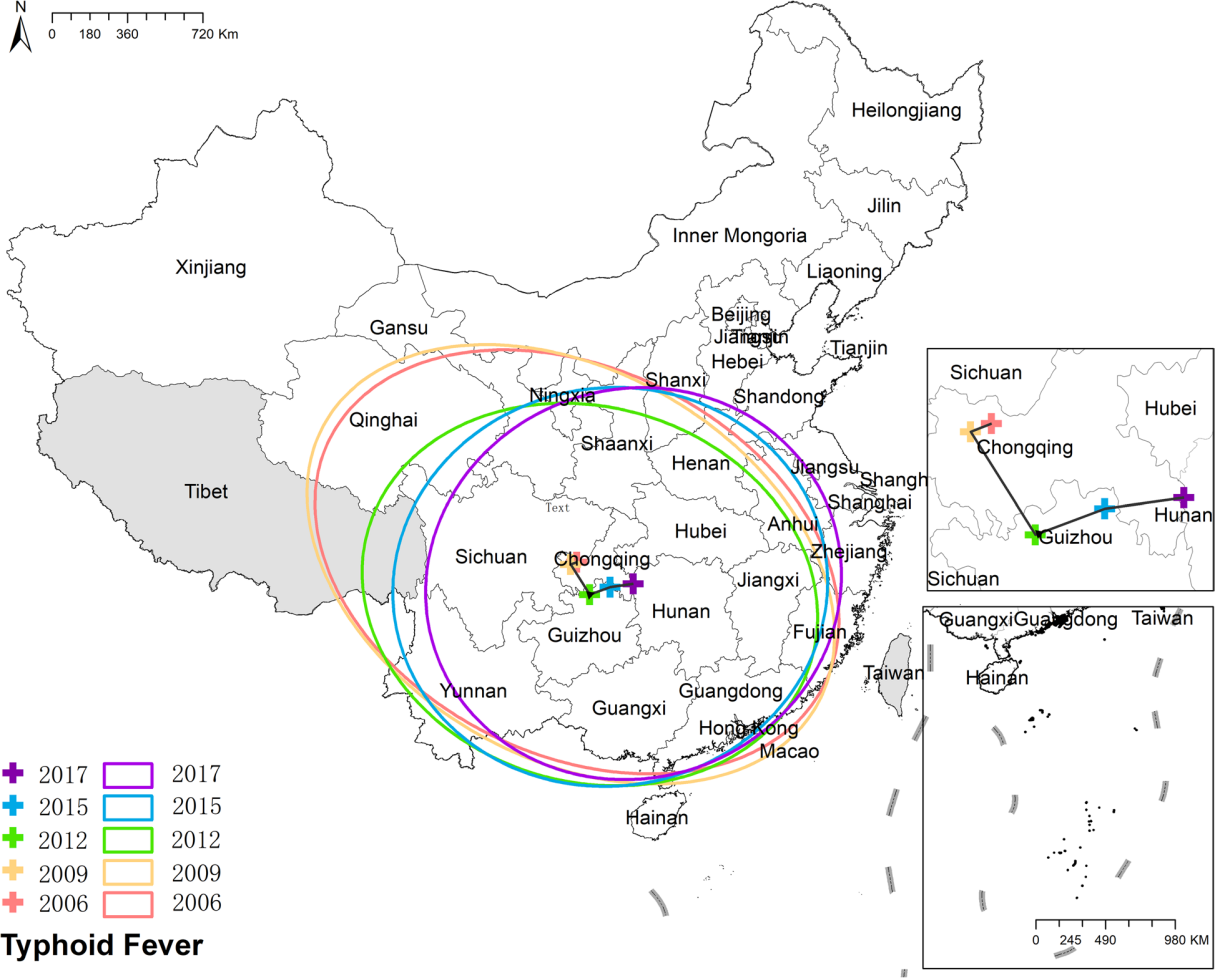
The results of mean center and standard deviational ellipse of typhoid fever

Initially, the mean center of typhoid was situated in Chongqing, after which it gradually shifted southwestward and eventually settled in Hunan province. Correspondingly, the ellipse of typhoid exhibited a similar movement. Initially, it displayed an elongation in the northwest-southeast direction, but over time, it gradually transformed into a more circular shape, signifying a reduction in directional characteristics. Additionally, the minor axes of the ellipse elongated, indicating a more dispersed distribution of typhoid fever. Figure 13 the results of mean center and standard deviational ellipse of typhoid fever.

#### Paratyphoid fever

**Fig. 14:**
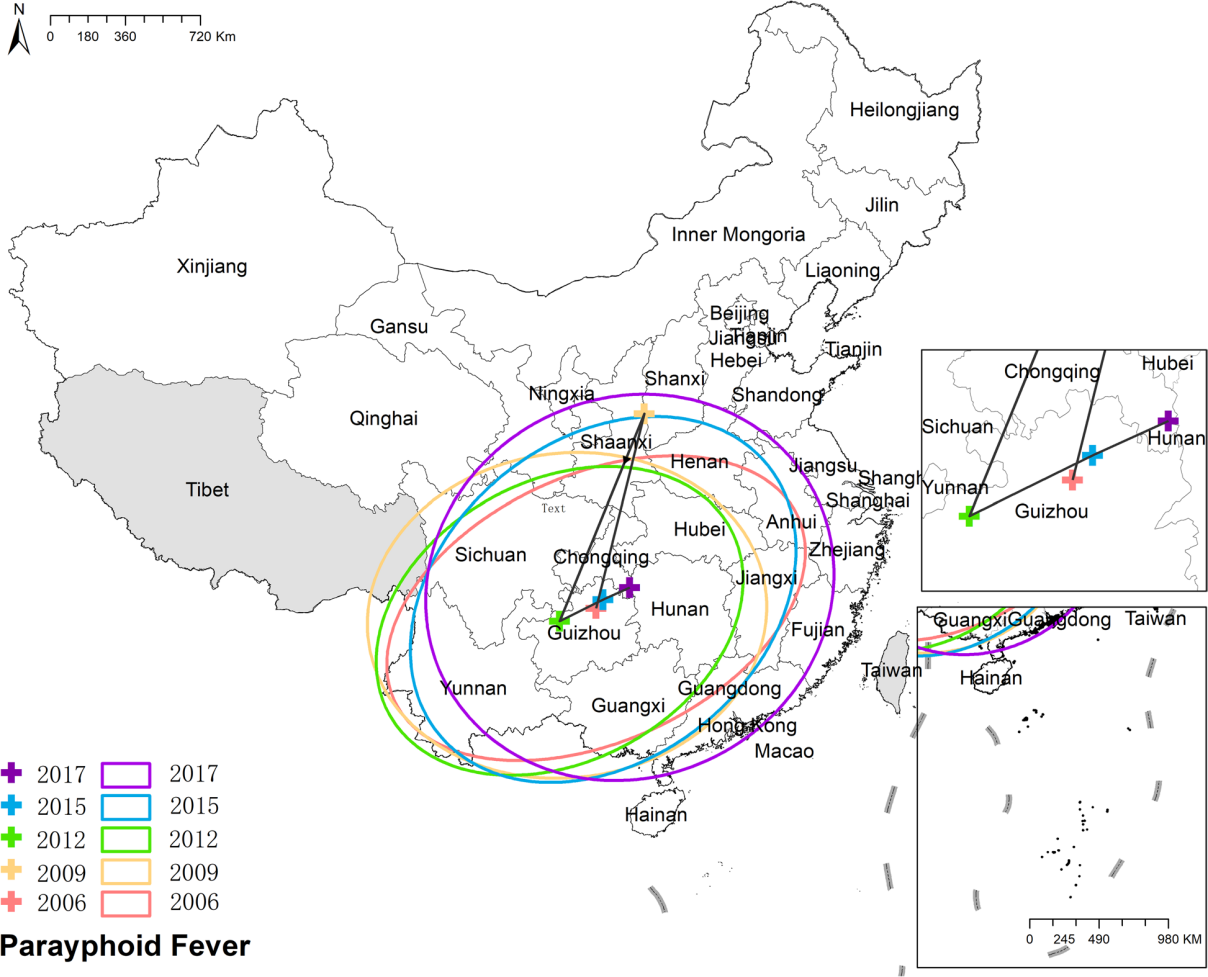
The results of mean center and standard deviational ellipse of paratyphoid fever

The mean center of paratyphoid remained predominantly within Guizhou province, with slight movements towards the northeast, except for the year 2009. Simultaneously, the corresponding ellipse gradually transitioned into a more circular shape and expanded, indicating a broader and more dispersed distribution of paratyphoid. Figure 14 the results of mean center and standard deviational ellipse of paratyphoid fever.

#### Bacillary dysentery

**Fig. 15:**
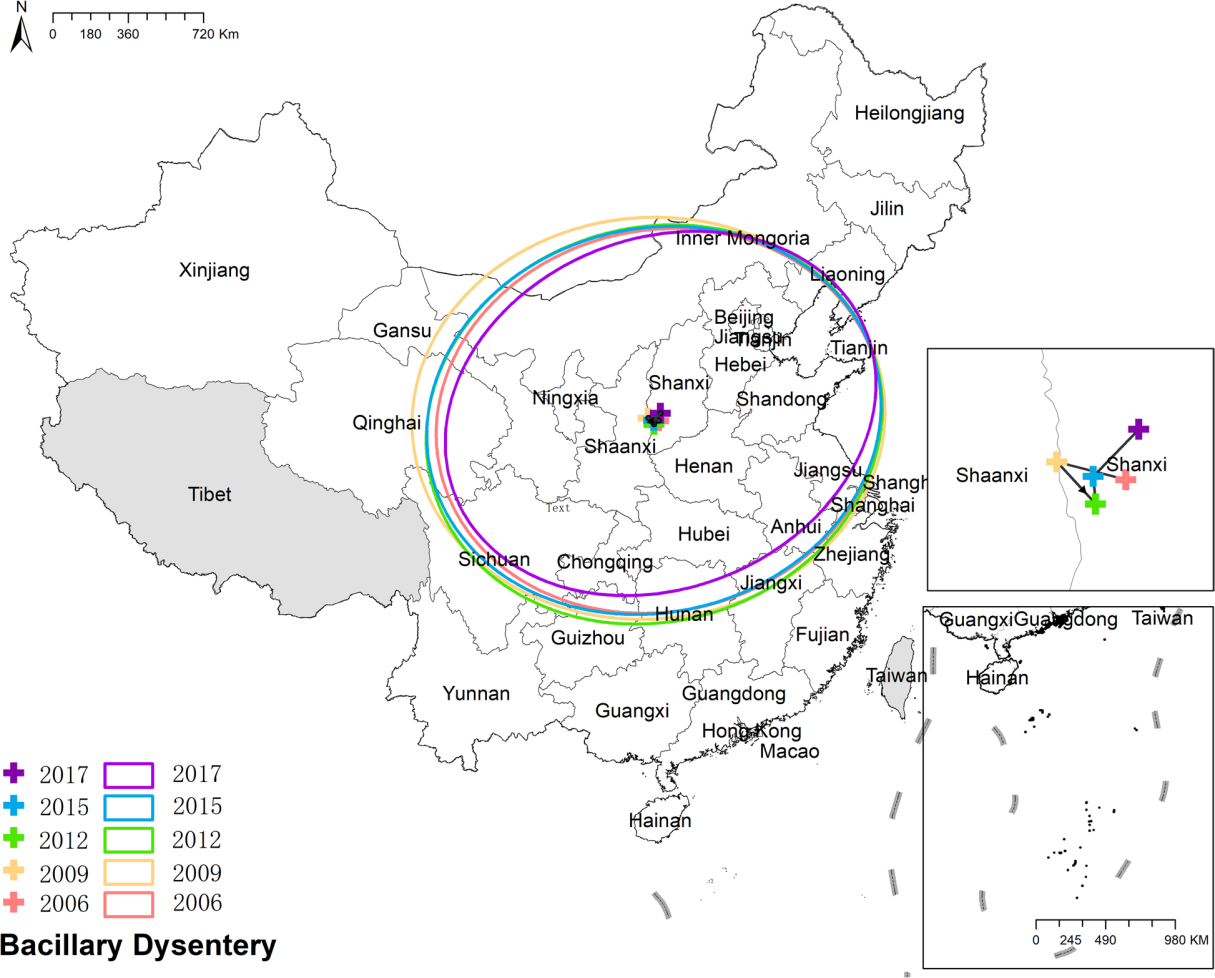
The results of mean center and standard deviational ellipse of bacillary dysentery

The mean center of bacillary dysentery remained consistently fixed within Shanxi province, which was reflected in the relatively stable circular shape of the corresponding ellipse. However, the minor axes of the ellipse became shorter over time. Furthermore, the shape of the ellipse exhibited an increasing elongation in the northeast-southwest direction, indicating a more concentrated and intense distribution of bacillary dysentery. Figure 15 the results of mean center and standard deviational ellipse of bacillary dysentery.

#### Amoebic dysentery

**Fig. 16:**
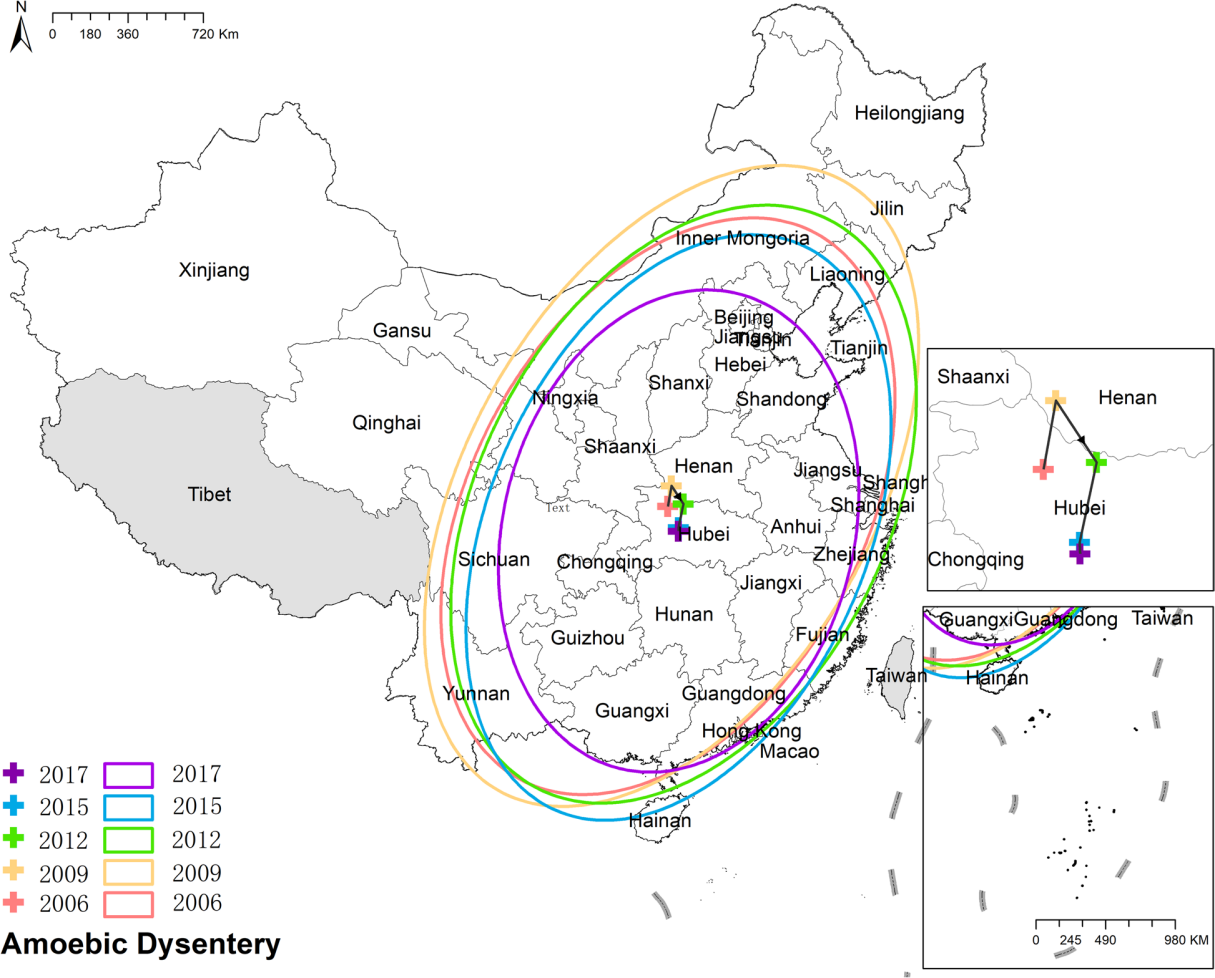
The results of mean center and standard deviational ellipse of amoebic dysentery

The mean center of amoebic dysentery initially shifted towards the north and subsequently moved towards the south, primarily concentrated within Hubei province. Notably, the corresponding ellipse displayed a clear elongation in the northeast-southwest direction and underwent a transformation towards a more north-south orientation. Moreover, the area encompassed by the ellipse decreased, indicating a more condensed and concentrated distribution of amoebic dysentery cases. Figure 16 the results of mean center and standard deviational ellipse of amoebic dysentery.

#### Hepatitis A

**Fig. 17:**
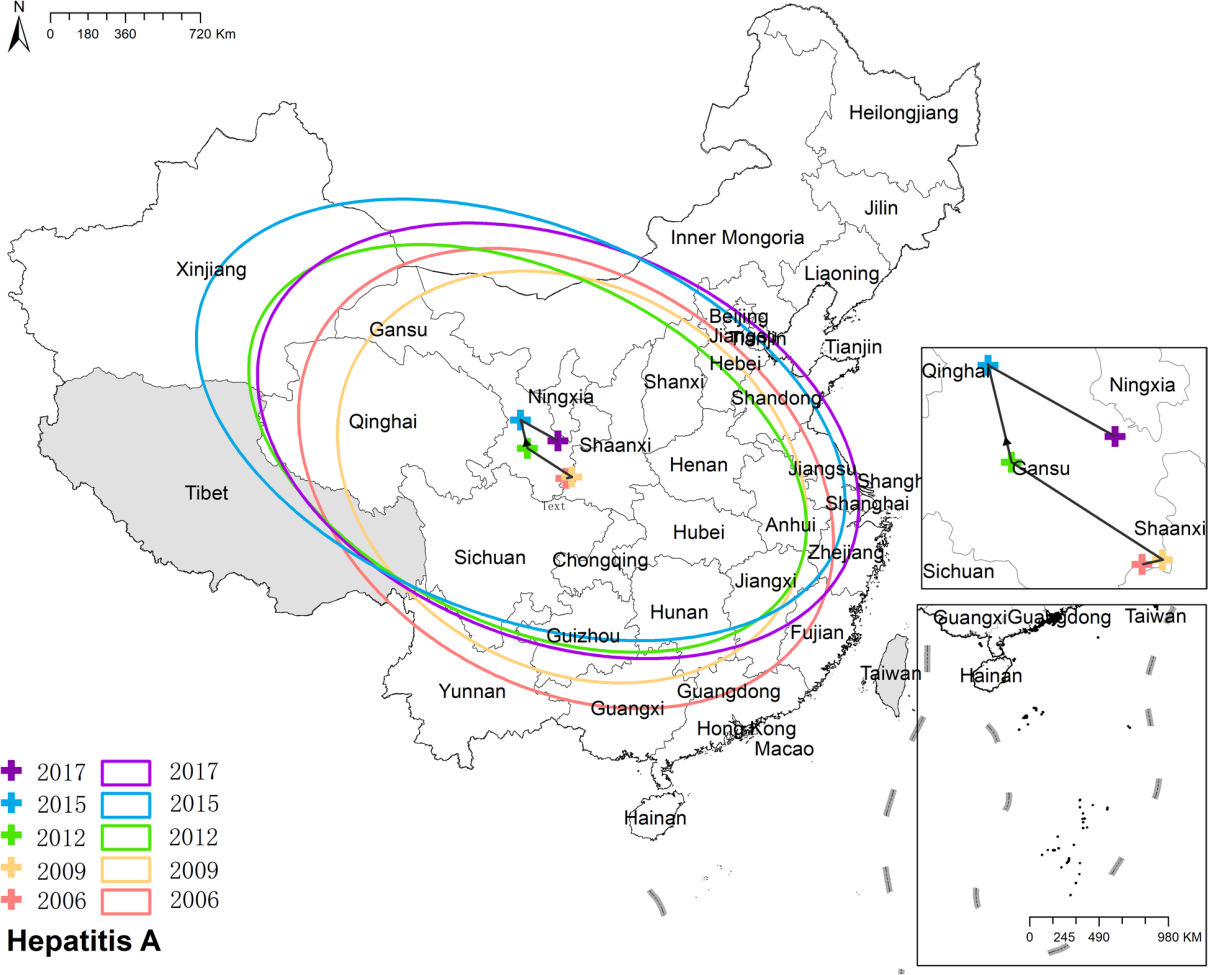
The results of mean center and standard deviational ellipse of hepatitis A

The mean center of hepatitis A initially shifted towards the northwest and subsequently returned to the southeast, ultimately locating in Gansu province. Notably, the major axes of the ellipse exhibited an elongation, indicating a more distinct northwest-southeast difference in the distribution of hepatitis A. Figure 17 The results of mean center and standard deviational ellipse of hepatitis A.

#### Hepatitis E

**Fig. 18:**
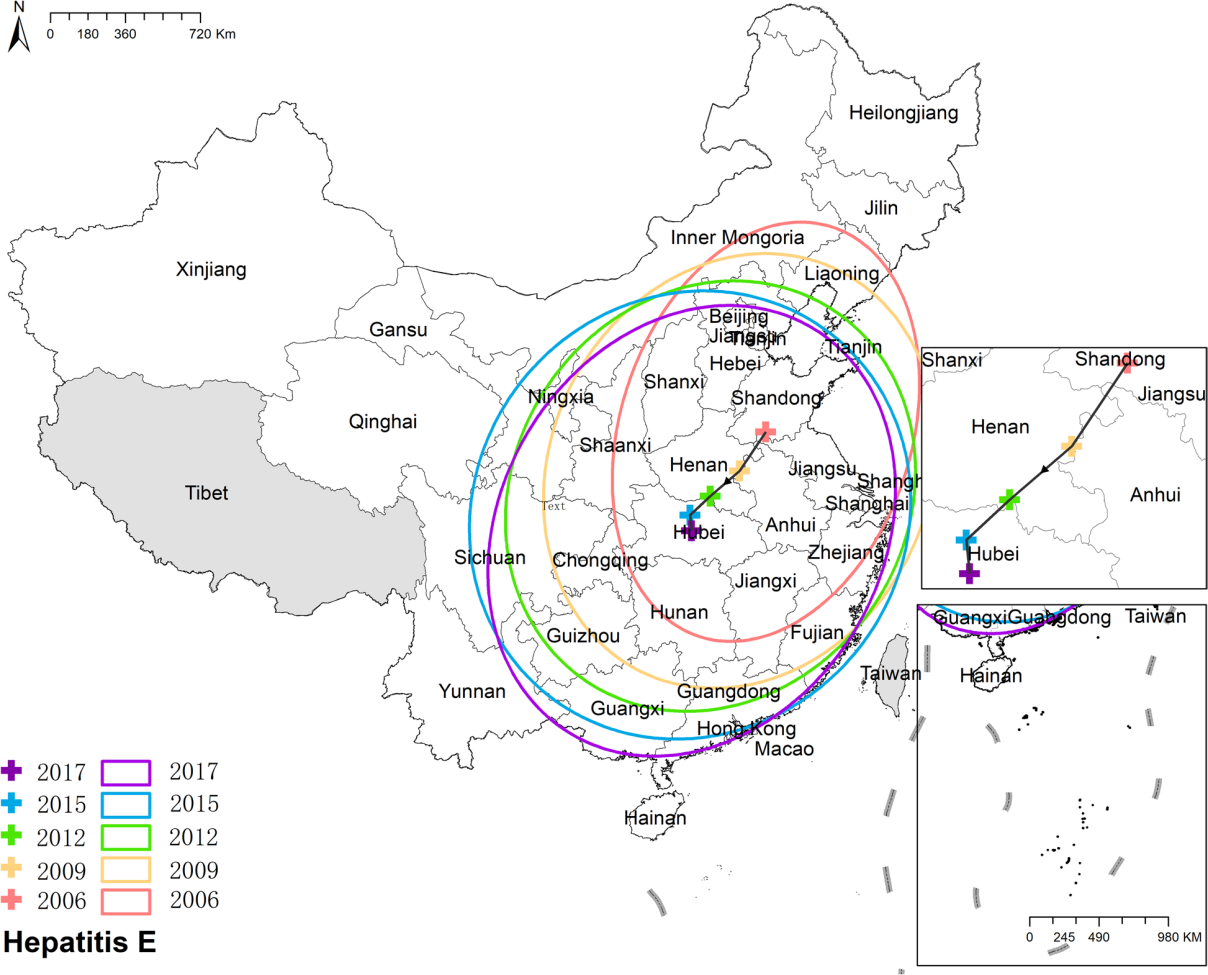
The results of mean center and standard deviational ellipse of hepatitis E

The mean center of hepatitis E initially resided in Shandong province before gradually shifting southwestward and ultimately settling in Hubei province. Concurrently, the ellipse representing its distribution transformed from an apparent northeast-southwest elongation to a more circular shape. Furthermore, the minor axes of the ellipse became longer, indicating a more dispersed and expanded distribution of hepatitis E. Figure 18 the results of mean center and standard deviational ellipse of hepatitis E.

#### Hand, Foot, and Mouth Disease (HFMD)

**Fig. 19:**
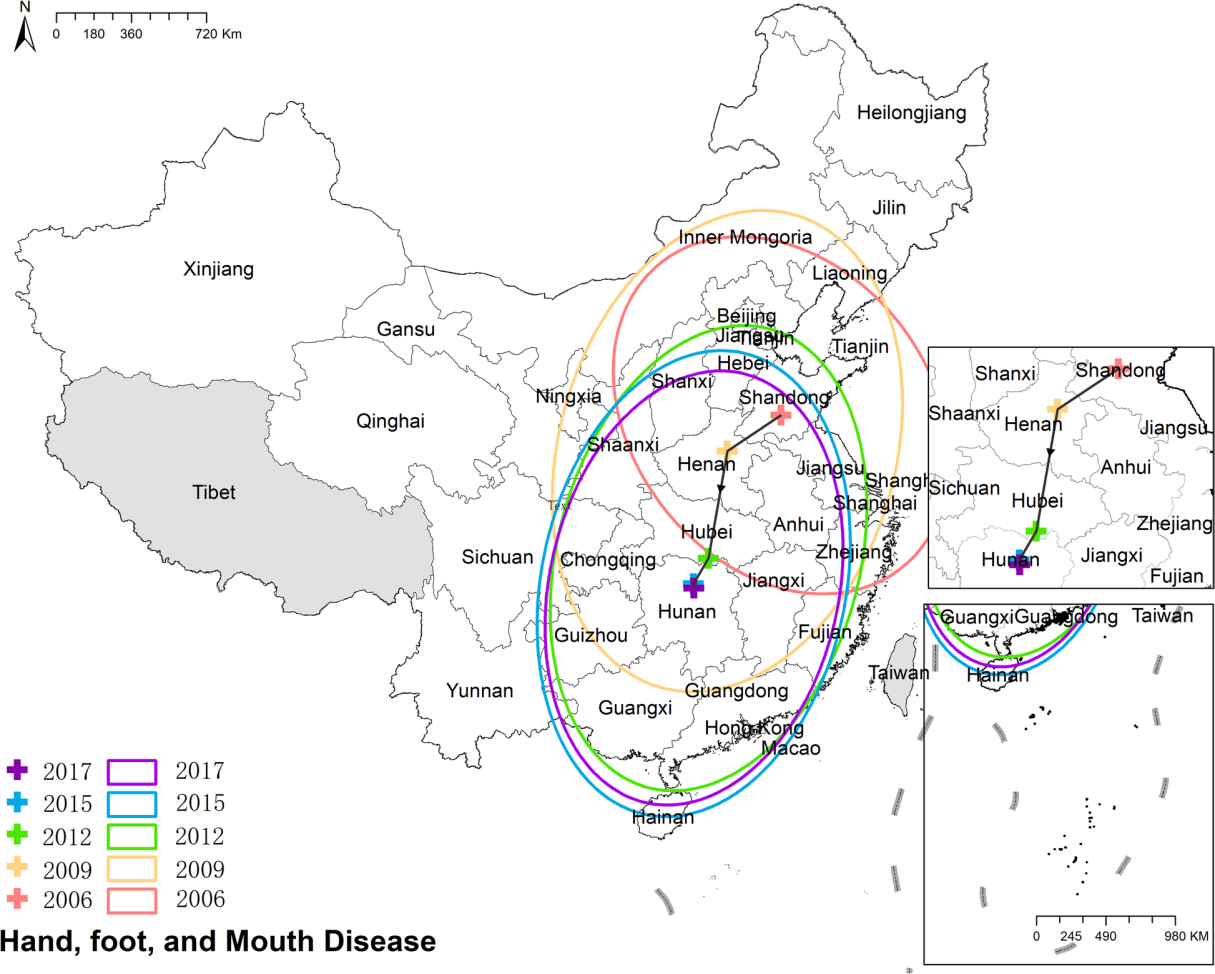
The results of mean center and standard deviational ellipse of hand, foot, and mouth disease

The mean center of HFMD exhibited a southwestward movement from Shandong province to Hunan province. Simultaneously, the shape of the ellipse transformed from a northwest-southeast elongation to a northeast-southwest elongation. This change in the shape of the ellipse signifies a shift in the difference of HFMD's distribution pattern. Figure 19 the results of mean center and standard deviational ellipse of hand, foot, and mouth disease.

#### Other infectious intestinal diseases

**Fig. 20:**
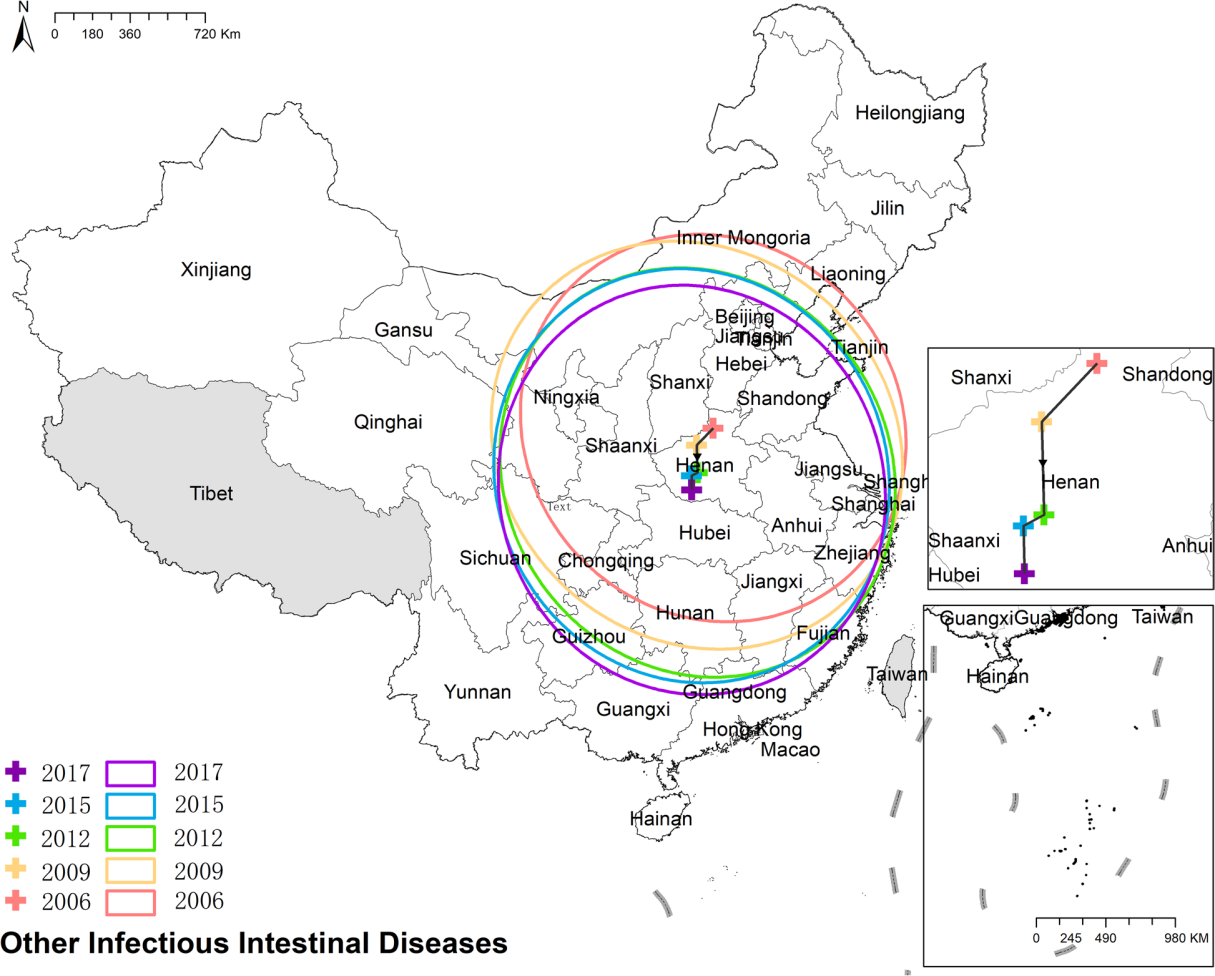
The results of mean center and standard deviational ellipse of other infecitous intestinal diseases

The mean center of the OIIDs (Other Intestinal Infectious Diseases) displayed a southwestward movement and remained primarily concentrated within Henan province. Although there was a slight change in the elongation direction of the ellipse, the overall shape of the ellipse was predominantly circular. This suggests that the directional characteristics of the OIIDs' distribution were not prominent. Figure 20 the results of mean center and standard deviational ellipse of other infecitous intestinal diseases.

### Exploration of influential factors

Table 2 presents the VIF results, demonstrating that all variables had VIF values below 10. As the result indicated the absence of concerning multicollinearity, we proceeded to include all the candidate variables in our model.

**Table 2:** Multicollinearity evaluation results

**Variable**	**VIF**
**ARHST**	3.54
**Urbanization**	1.45
**GDP**	1.36
**Population Density**	2.33
**Temperature**	3.71
**Precipitation**	6.98
**Humidity**	6.28
**Wind speed**	1.54

**Fig. 21:**
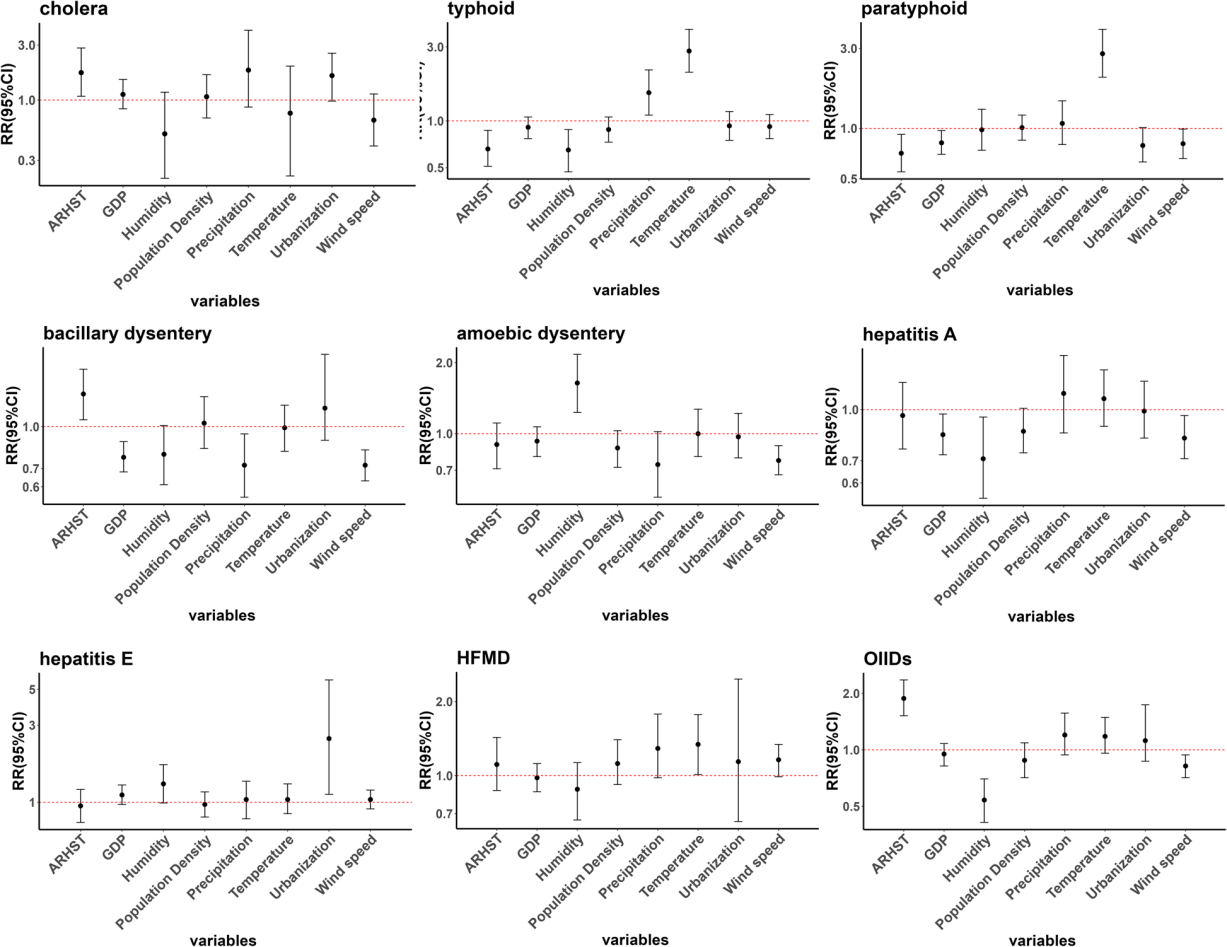
The estimated RR (95% CIs) of influential factors of different intestinal infections

Table 3 and Fig 21 show the estimated Relative Risk (RR) and 95% confidence intervals (CIs) based on the spatial-temporal interactive model. Since we have standardized all the variables before fitting the model, the interpretation of RR will be expressed based on one-Standard Deviation (SD) increase in the standardized covariates.

**Table 3:** The estimated RR (95% CIs) for influential factors of different intestinal infections

**Variable**	**Cholera**	**Typhoid fever**	**Paratyphoid fever**	**Bacillary dysentery**	**Amoebic dysentery**	**Hepatitis A**	**Hepatitis E**	**HFMD**	**OIIDs**
**ARHST**	1.73(1.08 - 2.83)*	0.66(0.51 - 0.87)*	0.71(0.55 - 0.92)*	1.32(1.06 - 1.63)*	0.90(0.71 - 1.11)	0.96(0.76 - 1.21)	0.95(0.75 - 1.20)	1.11(0.87 - 1.43)	1.88(1.52 - 2.36)*
**Urbanization**	1.63(0.98 - 2.55)	0.93(0.75 - 1.15)	0.79(0.63 - 1.01)	1.17(0.89 - 1.85)	0.97(0.79 - 1.22)	0.99(0.82 - 1.22)	2.48(1.12 - 5.72)*	1.14(0.65 - 2.47)	1.12(0.87 - 1.74)
**GDP**	1.12(0.84 - 1.51)	0.91(0.77 - 1.06)	0.82(0.70 - 0.97)*	0.77(0.68 - 0.88)*	0.93(0.80 - 1.07)	0.84(0.73 - 0.97)*	1.11(0.97 - 1.28)	0.98(0.86 - 1.12)	0.95(0.82 - 1.08)
**Population Density**	1.07(0.70 - 1.66)	0.88(0.73 - 1.06)	1.01(0.85 - 1.20)	1.03(0.83 - 1.29)	0.87(0.72 - 1.03)	0.86(0.74 - 1.01)	0.97(0.81 - 1.16)	1.12(0.92 - 1.40)	0.88(0.71 - 1.09)
**Temperature**	0.77(0.22 - 1.97)	2.82(2.06 - 3.89)*	2.79(2.02 - 3.90)*	0.99(0.81 - 1.20)	1.00(0.80 - 1.27)	1.08(0.89 - 1.32)	1.04(0.85 - 1.30)	1.34(1.01 - 1.77)*	1.18(0.96 - 1.49)
**Precipitation**	1.82(0.87 - 4.03)	1.52(1.09 - 2.13)*	1.07(0.80 - 1.46)	0.72(0.55 - 0.94)*	0.74(0.54 - 1.02)	1.12(0.85 - 1.46)	1.04(0.79 - 1.35)	1.29(0.98 - 1.78)	1.20(0.94 - 1.57)
**Humidity**	0.51(0.21 - 1.17)	0.65(0.47 - 0.88)*	0.98(0.74 - 1.30)	0.79(0.61 - 1.01)	1.64(1.23 - 2.17)*	0.71(0.54 - 0.95)*	1.30(0.99 - 1.71)	0.88(0.66 - 1.13)	0.54(0.41 - 0.70)*
**Wind speed**	0.67(0.40 - 1.13)	0.92(0.77 - 1.10)	0.81(0.66 - 0.99)*	0.72(0.63 - 0.82)*	0.77(0.67 - 0.89)*	0.82(0.71 - 0.96)*	1.04(0.91 - 1.19)	1.16(0.99 - 1.34)	0.82(0.71 - 0.94)*

*P≤0.05

The cholera incidence would increase by 73% for a 26.84% increase in ARHST. The typhoid fever incidence would increase by 182% for a 5.35℃ increase in temperature and by 52% for a 536.99 increase in precipitation. The typhoid fever incidence would decrease by 34% for a 26.84% increase in ARHST and by 35% for a 9.6% increase in humidity. The paratyphoid fever incidence would increase by 179% for a 5.35℃ increase in temperature. The paratyphoid fever incidence would decrease by 29%, 18%, and 19% for every SD increase in ARHST, GDP, and wind speed respectively. Bacillary dysentery would increase by 32% for a 26.84% increase in ARHST. The bacillary dysentery incidence would decrease by 23%, 28%, and 28% for every SD increase in GDP, precipitation, and wind speed. The amoebic dysentery incidence would increase by 64% for a 9.6% increase in humidity while decreasing by 23% for every SD increase wind speed. The hepatitis A incidence would decrease by 16%, 29%, and 18% for every SD increase in GDP, humidity, and wind speed. The hepatitis E incidence would increase by 148% for one SD increase in GDP. The HMFD incidence would increase by 34% for every SD increase in temperature. The OIIDs would increase by 88% for a 26.84% increase in ARHST while decreasing by 46% and 18% for every SD increase in humidity and wind speed.

## Discussion

To our knowledge, this study is the first to systematically show the influence of socioeconomic and meteorological factors on nine IIDs, across the vast region in mainland China. We showed the spatial and temporal distribution of IIDs incidence and found associations between factors and IIDs.

The descriptive results suggest that, during the study period, the occurrence of most IIDs has dramatically reduced, with uneven reductions in different diseases, while we observed increases in hepatitis E, HFMD, and other infectious intestinal diseases. That was consistent with a previous descriptive study on IIDs in China which found that class B IIDs were prevented with high efficiency while class C IIDs experienced a higher incidence rate and gradually increasing trend [13]. In developed countries, HAV infection incidence tends to decrease while HEV infections are increasingly observed, consistent with our findings as well.

Notably, throughout our study period, HFMD has been classified as a Class C infectious disease in China since 2008, mandating its reporting within 24 hours [14]. Consequently, the improvement in the surveillance system has resulted in an increase in the reported cases of HFMD. Additionally, the notifiable disease surveillance system network has undergone expansion, with gradual enhancements in diagnostic capacity, criteria, surveillance data reporting methods, and overall sensitivity [15]. As a result, there has been an overall increase in the reporting of other diseases, such as OIIDs. Therefore, it is crucial to consider these factors carefully when discussing and interpreting the findings of our study.

The findings suggested that ARHST had an inconsistent effect on IIDs. An increase in ARHST led to a decline in the occurrence of typhoid and paratyphoid, but an increase in morbidity of bacillary dysentery, and other intestinal infections. In addition, we found no significant effect of ARHST on the occurrence of cholera, amoebic dysentery, hepatitis A, hepatitis E, and HFMD. Empirical results of previous studies showed inconsistent effects as well. For example, in Jiangsu Province in China, a study found that the cumulative number of households using sanitary toilets was negatively associated with the aggregate annual incidence of class A and B IIDs but ineffectively with class C IIDs [2]. A study in Fiji found that sanitary conditions served as protective factors in the occurrence of typhoid fever [16]; Zuin found that low hygienic conditions are associated with high HAV and HEV seroprevalence [17]; besides, Knee et al. found their latrine intervention did not affect the bacterial intestinal infection in Mozambique [18]. Thus, findings from our study and others indicated that sanitary interventions might not be able to curb the spread of all kind of IIDs. However, a study found that latrine interventions have rarely been shown to prevent diarrhea, according to their historical review and meta-analysis. Their explanation for this conclusion was that environmental intervention and implementation contexts were too complex to average and required a better understanding of their effects [19]. This viewpoint might help us to explain our result. Due to the limitation of our ecological study, we averaged the effect of ARHST based on the unit of the province, which could mask the crucial environmental differences.

Urbanization led to an increase in the occurrence of hepatitis E and showed no significant effect on the occurrence of the rest IIDs in our findings. However, empirical results in previous studies tended to regard urbanization as a protective factor. For example, Masoud et al. found the protective effects of urbanization on typhoid fever in Iran [11], and Ceran et al. found urbanization associated with the reduction of hepatitis A seropositivity in Turkey [20]. It might be the lack of other related confounders like male sex and underlying conditions [21] that caused this inconsistency. Another explanation was that urbanization could be related to the increase in consumption of pork or other meat products [22], as zoonotic transmissions of hepatitis E were increasingly observed [23]. Nevertheless, few studies conformed with our results. A study in Australia also found no statistically significant association between urbanization and the prevalence of infectious gastroenteritis illness [24]. Jiang et al. studied the urbanization effect on population health in China and found that when GDP reached a certain threshold, it would reduce the health-prompting effect of urbanization in inland and northern provinces. [25] Therefore, our result on urbanization could be the consequence of this reduction effect by GDP. For GDP, we observed a negative association between GDP and morbidity of paratyphoid fever, bacillary dysentery, and hepatitis A, and no significant association between GDP and morbidity of the rest IIDs. Some previous studies assessed the protective effect of GDP on IIDs. Zuin found that socioeconomic level is negatively associated with HAV seroprevalence [17]. Masoumi et al. identified socioeconomic status as the key determinant in the downfall of typhoid cases [26]. However, a study in Guangzhou province in China found no significant effect of GDP on typhoid [27], in accord with our result of typhoid. The further interpretation was that socioeconomic status does not affect the health of all residents equally, especially when our study units were as big as a province. Nevertheless, we did confirm the protective effect of GDP on some IIDs, pointing forward to future intervention.

In the study, a significant effect of population density on the occurrence of all IIDs was not found. However, it is worth noting that previous studies have reported inconsistent results in this regard. For instance, Du found a positive association between higher population density and increased incidence of HFMD in Guangzhou province [28]. Similarly, Xu identified population density as an important determinant of bacillary dysentery in the Beijing-Tianjin-Hebei region [29]. One plausible explanation for the disparity in findings could be the scale at which the studies were conducted. The study areas in both Du and Xu's research were based on smaller spatial units within the province, allowing for a more detailed analysis of population density effects. In contrast, our study employed larger provincial units, potentially averaging out the effects of population density, which may vary significantly within each province, particularly between urban and rural areas.

The results exhibited a significant effect of meteorological factors on IIDs as well. According to previous studies, there are various postulations on the way meteorological factors affect the occurrence of IIDs that is via microorganism activity and human behavior. Based on these assumptions, the reason why meteorological variables had different effects on IIDs was that the sensitivity to changes in heat, moisture, nutrients, and related conditions was different in pathogens [30].

In findings, a temperature rise elevated the incidence of typhoid, paratyphoid, and HFMD, consistent with previous studies that identified that warmer climate conditions would lead to the increase of HFMD [31], diarrhea [32] and typhoid [33] and paratyphoid fever [33]. Warmer conditions might increase bacterial pathogen loads in animal reservoirs and prolong transmission seasons [34]. Higher temperature also leads to an increase in food spoilage and change in the behavior of hosts, like altering consumption practices and gathering in air-conditioned public places [35].

Precipitation was considered a risk factor in cholera and typhoid but a protective factor in bacillary dysentery in our results. In previous studies, high precipitation acted as a risk factor for IIDs [36], as it was conducive to contaminating water, fruits, and vegetables with feces. These empirical results were consistent with our findings on cholera and typhoid but contradictory to bacillary dysentery. The probable interpretation was the “dilution effect” brought by Levy et al. that when rainfall lasts long, it might dilute microbial concentrations [37].

We observed a negative association between humidity and cholera, typhoid, hepatitis A, and other intestinal infectious diseases and a positive association between humidity and amoebic dysentery, discrepant with previous studies in Yunnan and Kolkata. Kim et al. found that enteric viruses and HAV became inactive in higher relative humidity levels, which might help explain our findings [38].

We observed a negative association between wind speed and cholera, paratyphoid, bacillary dysentery, amoebic dysentery, hepatitis A, and other intestinal infectious diseases. Few studies found a significant association between wind speed and IIDs, except for a study in Beijing that found a similar negative association between wind speed and bacillary dysentery [3]. Due to the lack of related studies, we could not interpret the detailed mechanism of how wind speed affects the transmission between pathogens and hosts. However, we noticed a positive association between wind speed and HFMD. Though, unlike other IIDs, the result conformed with a study in Hongkong [39]. The probable reason might be the different transmission of HFMD and the rest IIDs. Since the droplet transmission of HFMD, Ma et al. reckoned that wind speed might favor the spread of disease through respiratory droplets.

There are some limitations to consider in this study. First, our data is in the annual and provincial units where the averaged data could not accurately reflect the causal association between determinants and diseases. Second, our research is based on provincial units, which are administrative divisions. However, due to the vast geographic expanse of the Chinese mainland, it encompasses several climatic zones. We propose the possibility of conducting research based on these climatic zones or subdividing our model into multiple climatic zones. This approach would enable us to perform a more detailed analysis of meteorological variables and examine their relationships within different climatic zones. Moreover, the associations between our factors and IIDs were complex and involved various confounders. The variables included in this study were limited, for example, the absence of food consumption data. Thus, we hope to obtain more related variables in our future study. In general, due to the limitation of ecological study, our results are not reliable enough to build solid causal inferences. Therefore, future studies should base on the individual level and consider more rigorous research.

## Conclusions

In conclusion, this study systematically and quantitatively studied the effect of socioeconomic and meteorological factors on IIDs, which provided causal clues for future studies and guided government planning.

## Data Availability

The datasets supporting the conclusions of this article are available in the Chinese National Bureau of Statistics (https://data.stats.gov.cn), the Chinese National Bureau of Statistics (https://data.stats.gov.cn) and the Global Historical Climatology Network-Daily (Version 3) in US National Centers for Environmental Information (https://www.ncei.noaa.gov).

## Abbreviations

ARHST: Access Rate to Harmless Sanitary Toilets
Cis: Confidence Intervals
GDP: Gross Domestic Product
HAV: Hepatitis A
HEV: Hepatitis E
HFMD: Hand, Foot, and Mouth Disease
IIDs: Intestinal Infectious Diseases
OIIDs: Other Infectious Intestinal Diseases
SD: Standard Deviation
VIF: Variance Inflation Factor
ZIP: Zero-Inflated Poisson

## References

[1] Chinese Center for Disease Control and Prevention https://www.phsciencedata.cn Accessed 1 Feb 2023

[2] Chen T, Kallawicha K (2021). Association between sanitary toilet coverage rate and intestinal infectious disease in Jiangsu province, China. Sci Rep..

[3] Li ZJ, Zhang XJ, Hou XX, Xu S, Zhang JS, Song HB (2015). Nonlinear and threshold of the association between meteorological factors and bacillary dysentery in Beijing China. Epidemiol Infect.

[4] Wikle CK, Berliner LM, Cressie N (1998). Hierarchical Bayesian space-time models. Environ Ecol Stat..

[5] Cao K, Yang K, Wang C, Guo J, Tao L, Liu Q, et al. Spatial-temporal epidemiology of tuberculosis in Mainland China: an analysis based on Bayesian theory. Int J Environ Res Public Health. 2016;13(5):469. 10.3390/ijerph13050469 PMC4881094 27164117

[6] Liu F, Zhang Z, Chen H, Nie S (2020). Associations of ambient air pollutants with regional pulmonary tuberculosis incidence in the central Chinese province of Hubei: a Bayesian spatial-temporal analysis. Environment Health..

[7] Lambert D (1992). Zero-Inflated Poisson Regression, with an application to defects in manufacturing. Technometrics..

[8] Ghosh SK, Mukhopadhyay P, Lu J-C (2006). Bayesian analysis of zero-inflated regression models. J Stat Plan Inference..

[9] Neelon BH, O'Malley AJ, Normand SL (2010). A Bayesian model for repeated measures zero-inflated count data with application to outpatient psychiatric service use. Stat Modelling..

[10] Neelon B, Chang HH, Ling Q, Hastings NS (2016). Spatiotemporal hurdle models for zero-inflated count data: Exploring trends in emergency department visits. Stat Methods Med Res..

[11] Masinaei M, Eshrati B, Yaseri M (2020). Spatial and spatiotemporal patterns of typhoid fever and investigation of their relationship with potential risk factors in Iran, 2012–2017. Int J Hygiene Environment Health..

[12] Franke GR. Multicollinearity. Wiley International Encyclopedia of Marketing 2010.

[13] Mao Y, Zhang N, Zhu B, Liu J, He R (2019). A descriptive analysis of the Spatio-temporal distribution of intestinal infectious diseases in China. BMC Infect Dis..

[14] China NHCotPsRo. Notice of the Ministry of Health on the inclusion of hand, foot and mouth disease in the management of statutory infectious diseases.http://www.nhc.gov.cn/wjw/gfxwj/201304/587bb99411f4491da0b76eeab9d79b01.shtml Accessed 13 Sept 2023

[15] Wang L, Ren X, Cowling BJ, Zeng L, Geng M, Wu P, et al. Systematic review: National notifiable infectious disease surveillance system in China. Online J Public Health Inform. 2019;11(1):e414.

[16] Jenkins AP, Jupiter SD, Jenney A, Naucukidi A, Prasad N, Vosaki G, et al. Environmental foundations of typhoid fever in the Fijian residential setting. Int J Environ Res Public Health. 2019;16(13):2407. 10.3390/ijerph16132407 PMC6651141 31284613

[17] Zuin M, Caserta C, Romanò L, Mele A, Zanetti A, Cannatelli R (2017). Seroepidemiology of HEV and HAV in two populations with different socio-economic levels and hygienic/sanitary conditions. Eur J Clin Microbiol Infect Dis..

[18] Knee J, Sumner T, Adriano Z, Anderson C, Bush F, Capone D, et al. Effects of an urban sanitation intervention on childhood enteric infection and diarrhea in Maputo, Mozambique: a controlled before-and-after trial. Elife. 2021;10:e62278. 10.7554/eLife.62278 PMC8121544 33835026

[19] Contreras JD, Eisenberg JNS. Does basic sanitation prevent diarrhea? Contextualizing recent intervention trials through a historical lens. Int J Environment Res Public Health. 2020;17(1):230. 10.3390/ijerph17010230 PMC6981821 31905628

[20] Ceran N, Yüksel Kocdogan F, Mert D, Erdem I, Dede B, Adaleti R (2012). Hepatitis A seroprevalence in children and young adults in Istanbul, Turkey: seroprevalence change and associated factors. J Viral Hepat..

[21] FitzSimons D, Hendrickx G, Vorsters A, Van Damme P (2010). Hepatitis A and E: update on prevention and epidemiology. Vaccine..

[22] Fenaux H, Chassaing M, Berger S, Gantzer C, Bertrand I, Schvoerer E (2019). Transmission of hepatitis E virus by water: an issue still pending in industrialized countries. Water Res..

[23] Hartard C, Gantzer C, Bronowicki J-P, Schvoerer E (2019). Emerging hepatitis E virus compared with hepatitis A virus: a new sanitary challenge. Rev Med Virol..

[24] Hall GV, Kirk MD, Ashbolt R, Stafford R, Lalor K (2006). Frequency of infectious gastrointestinal illness in Australia, 2002: regional, seasonal and demographic variation. Epidemiol Infect..

[25] Jiang T-B, Deng Z-W, Zhi Y-P, Cheng H. Gao Q. The effect of urbanization on population health: evidence from China. Front Public Health; 2021. p. 9. 10.3389/fpubh.2021.706982 PMC8242255 34222193

[26] Masoumi Asl H, Gouya MM, Nabavi M, Aghili N (2013). Epidemiology of typhoid fever in Iran during last five decades from 1962–2011. Iran J Public Health..

[27] Wang Y, Lai Y, Du Z, Zhang W, Feng C, Li R, et al. Spatiotemporal distribution of hand, foot, and mouth disease in Guangdong Province, China and potential predictors, 2009-2012. Int J Environ Res Public Health. 2019;16(7):1191. 10.3390/ijerph16071191 PMC6480297 30987085

[28] Du Z, Yang B, Jalaludin B, Knibbs L, Yu S, Dong G (2022). Association of neighborhood greenness with severity of hand, foot, and mouth disease. BMC Public Health..

[29] Xu C, Li Y, Wang J, Xiao G (2017). Spatial-temporal detection of risk factors for bacillary dysentery in Beijing, Tianjin and Hebei, China. BMC Public Health..

[30] Levy K, Woster AP, Goldstein RS, Carlton EJ (2016). Untangling the impacts of climate change on waterborne diseases: a systematic review of relationships between diarrheal diseases and temperature, rainfall, flooding, and drought. Environment Sci Technol..

[31] Jiang X, Ma Y, Lv Q, Liu Y, Zhang T, Yin F (2023). Influence of social and meteorological factors on hand, foot, and mouth disease in Sichuan Province. BMC Public Health..

[32] Alexander KA, Carzolio M, Goodin D, Vance E. Climate change is likely to worsen the public health threat of diarrheal disease in Botswana. Int J Environment Res Public Health. 2013; 10(4): 1202-30. 10.3390/ijerph10041202 PMC3709313 23531489

[33] Gao Q, Liu Z, Xiang J, Zhang Y, Tong MX, Wang S (2021). Impact of temperature and rainfall on typhoid/paratyphoid fever in Taizhou, China: effect estimation and vulnerable group identification. Am J Trop Med Hyg..

[34] Lal A, Hales S, French N, Baker MG (2012). Seasonality in human zoonotic enteric diseases: a systematic review. PLoS One..

[35] Huang Y, Deng T, Yu S, Gu J, Huang C, Xiao G (2013). Effect of meteorological variables on the incidence of hand, foot, and mouth disease in children: a time-series analysis in Guangzhou. China. BMC Infect Dis..

[36] Ghazani M, FitzGerald G, Hu W, Toloo GS, Xu Z. Temperature variability and gastrointestinal infections: a review of impacts and future perspectives. Int J Environ Res Public Health. 2018;15(4):766. 10.3390/ijerph15040766 PMC5923808 29659519

[37] Levy K, Hubbard AE, Eisenberg JN (2009). Seasonality of rotavirus disease in the tropics: a systematic review and meta-analysis. Int J Epidemiol..

[38] Kim SJ, Si J, Lee JE, Ko G (2012). Temperature and humidity influences on inactivation kinetics of enteric viruses on surfaces. Environ Sci Technol..

[39] Ma E, Lam T, Wong C, Chuang SK (2010). Is hand, foot and mouth disease associated with meteorological parameters?. Epidemiol Infect..

